# Supplementary material for: A change in cis-regulatory logic underlying obligate versus facultative muscle multinucleation in chordates
Source: Development. 2024 Sep 3;151(20):dev202968. doi: 10.1242/dev.202968 (PMC11441980; doi:10.1242/dev.202968)
Supplement: Supplementary information [file develop-151-202968-s1.pdf]

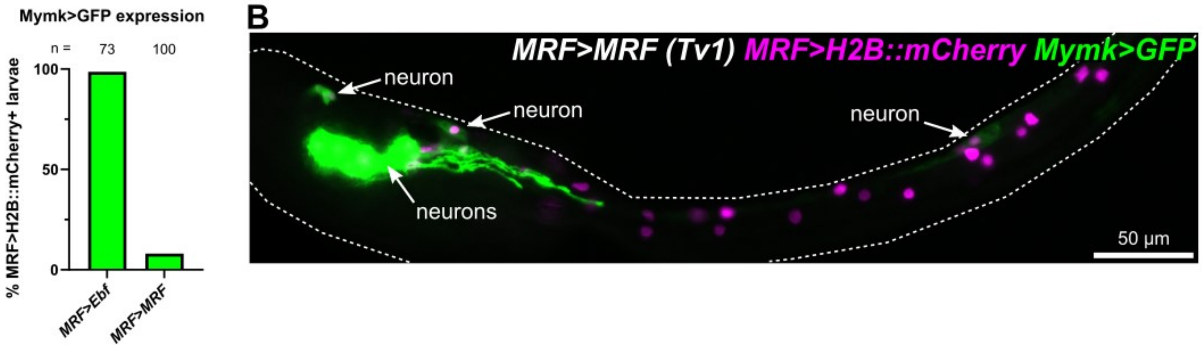

Fig. S1. Increased MRF dose in larval tail muscles does not adequately replace Ebf (**A**) Scoring of larvae at 21 hours post-fertilization (hpf) comparing effect of Ebf or MRF overexpression on ectopic *Mymk>GFP* expression in larval tail muscles ($p < 0.0001$). (**B**) Most of the effect of *MRF>MRF* on activating ectopic *Mymk>GFP* was limited to the nervous system, likely due to leaky activity of the *MRF* promoter in Ebf+ neurons. See **Table S1** for statistical test details.

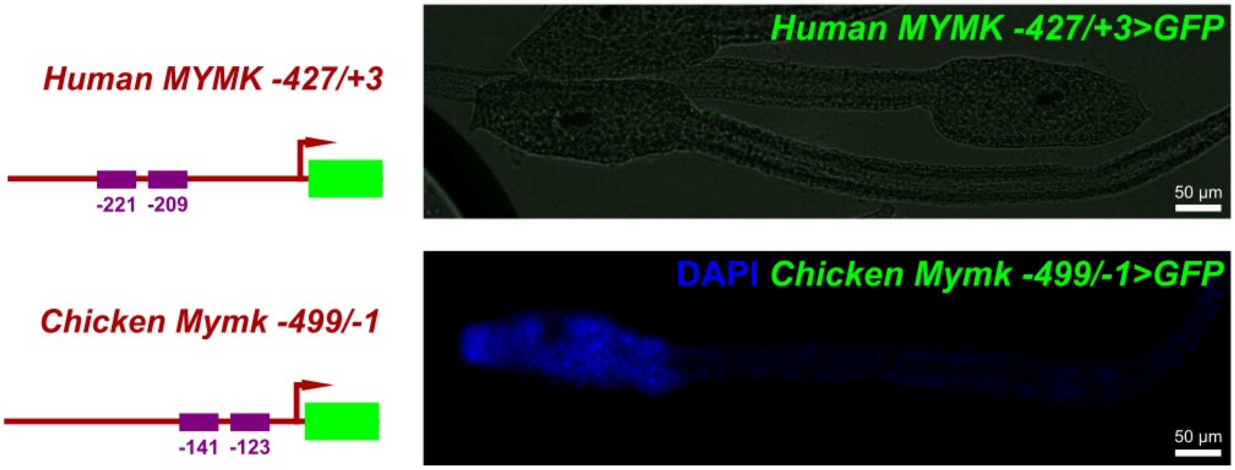

Fig. S2. Human *MYMK* and chicken *Mymk* reporter plasmids are not active in *Ciona* Left: diagrams describing human *MYMK* and chicken *Mymk* reporter plasmids using previously published sequences (Luo et al., 2015; Zhang et al., 2020) and their predicted MRF family member binding sites (purple boxes). Size/spacing of sites is not to exact scale. See **Supplemental Sequences File** for detailed sequences. Right: images of larvae at 17 hours post-fertilization (hpf) electroporated with the reporter plasmids at left, showing no expression in larval tail muscles.

Development • Supplementary information

MRF binding site optimization

wild type sites		
JASPAR PWM	MRF-152	MRF-136
MA0499.1.Myod1	-0.37	13.07
MA0499.2.MYOD1	2.79	9.57
MA0500.1.Myog	-0.47	13.65
MA0500.2.MYOG	8.47	11.15
Average	2.61	11.86

TCCAGCTGAATCAGACAACAGCTGAA
-152 -136

"optimized" sites		
JASPAR PWM	MRF-152	MRF-136
MA0499.1.Myod1	13.56	13.86
MA0499.2.MYOD1	10.50	10.25
MA0500.1.Myog	13.59	14.24
MA0500.2.MYOG	13.56	13.99
Average	12.80	13.09

GGCAGCTGGGTCAGACGGCAGCTGGG
-152 -136

Ebf binding site optimization

wild type site	
JASPAR PWM	Ebf-116
MA0154.3.EBF1	11.49
MA1604.1.Ebf2	8.32
MA1637.1.EBF3	7.90
Average	9.24

TTCCCAAGAT

"optimized" site	
JASPAR PWM	Ebf-116
MA0154.3.EBF1	15.03
MA1604.1.Ebf2	14.16
MA1637.1.EBF3	14.29
Average	14.49

CCCCCAGGGG

Fig. S3. JASPAR scores before and after "optimization" of putative binding sites Predicted JASPAR scores for affinity of MRF (top) or Ebf (bottom) to their respective putative binding sites, before and after point mutations to "optimize" them, or rather increase their predicted JASPAR scores. Predictions based on individual human transcription factor PWMs and their averages shown.

Table S1. Scoring data for *Ciona* experiments and statistical test details

Available for download at
https://journals.biologists.com/dev/article-lookup/doi/10.1242/dev.202968#supplementary-data

Table S2. DESeq2 analysis of differential gene expression of Ebf overexpression in developing larval tail muscles measured by Illumina bulk RNAseq

Available for download at
https://journals.biologists.com/dev/article-lookup/doi/10.1242/dev.202968#supplementary-data

Table S3. Comparison of genes significantly up- or down-regulated in the RNAseq analysis in the current study and the microarray study of Razy-Krajka et al. 2014

Available for download at
https://journals.biologists.com/dev/article-lookup/doi/10.1242/dev.202968#supplementary-data

Table S4. Predicted JASPAR affinity scores for putative MRF and Ebf sites in the *Ciona Mymk* promoter using various human ortholog position weight matrices

Available for download at
https://journals.biologists.com/dev/article-lookup/doi/10.1242/dev.202968#supplementary-data

Supplementary Materials and Methods

Relevant coding and non-coding sequences:

```
>Ciona Mymk promoter (-508/-1), from Zhang et al. 2022
TGCTCTGGAAAATTTACCAAGGGAAACTCCCTCACGTGGTAACATAGAAATAGTACAAAAAATGCTTAAA
ATTGTTTTTCACCAAAACTAACGGTAcACATACCGTTGCCTAAACTAGGCAAAACACATATTAGATTGCA
TTTAAACTGGTAAATGTCCTAATAATTTAAAACTTGCATTAACAATAAATTTAGTTGCCAATAATATAAC
TTTTAAATTGCGTATTTTAAGTATGTACATAGTTAAGAATATTTATTCGGGCCATAAATGTGTATTTTAA
AGTTGTTTATATTTAGACCAAATACTGATATTTTAATAAACGTTTTGGGTCGTGAGAAATATCACATTAT
TGTGTCCAGCTGAATCAGACAACAGCTGAAGACCACATGCCAATTCCCAAGATAATGGTGCAGCACGCGC
AAAACAACCGAGAGTACCGTAGTACATTTGGTATGTCGCCTTTAGAGCATTCATTGCATAGTGTTAAACC
GTAGATTAAATCATAGTT
```

```
>Ciona MRF promoter (-2604/-1), from Stolfi et al. 2015
gcaagctcctttggggtttggccactcggtcggcaagtaaaatatcatttaagtttttaaatgtcgttta
aatggcagcagccgtaacaggtggactgccacgtgtcgcccggtgtgatcgtgtttccatcccaagggtg
gtagtgcaggcatgccgacgctcatatgagccattcataataatttggcgcatgtaagaggaaaatgcac
cagtaagatggtgccagcacattgtaattaaaatgtggtgctacggagaagtgtggaaatcgttatagga
aataaaatcccttattcccgatcgtttttaccaactaacgacgttattttatatttgcgaagttacggtt
acataattatagtagggtgggggaagatggaacaactttagcacagaatatataaatatcctgatcgagt
tctaaacaattaacaatggtccatgggttgcgaagatacggttttataattctttgaatgttctttgttt
gctaccaaacgggacgagaaaagaaattaaagcatgaaccattttacttcaacctactactacagtatct
aagtaatttcttttcgaaacagatttaacttctttgcgatttcaattagcgaatcccagtttgaccgtta
aagctatagcttagagtacgtggtgctaaatttcgttgattcgcgcccctgaagcgagcaacttattaga
tagaaatcccagctggtaacaacatgtgttcatcgccctactgatgtctgtggttgccgaactctcttta
cgagactgagagccgacgtctcgataaacgaggttgtgtattatgatttatttaggaaataaaaagtaag
aaaataaaaatttaattaagtaaaatacaaacgtttacaaaaattatgaatttttttcaccaatttttaa
ttttattcaagtatccgacgacgtttaagaactttaaaatccaaatcctttgctgcttcctagaattata
ctgtggggtaagatggaaaccgtaaatattctgtgtttacaaccaaataggacgataaaagagaatgaaa
cgtggacaatttgccctaacacattacacggctaaaaatattaggtttcctcctaaaatagcagcgacga
ccataaggttattcgaagatgaaaggccaaagtaacggatgcaagtttttgcaagacaatgctgtaattg
ttacgtcacagaggaattccacaacttattccagagcagacgagcgtcgcgtttactgtttgctaaaaat
ggcgactcgaggtgacaattacaattcgcgctgcgaatgacgtaataatgctattcgctgtgatgaaata
atagaagtggaaattcgaacaataccaacccacgatgaagcatatgtagcgaatccggaattaaacctct
agacttgcttgggaaatgcatggcgtatatgagtgtattacgtgatcgttaaagcgtcttatacagtcgc
attagaagctattttaagaaaatccacggcccctcatgcaaatatagctgtgatctaataggttaggtta
gtttacattaagtttaataatgtcgatcacttattgcatgtttcatgatcttgcaaatatgatgacgttg
tatattgaggttgctgctttcagttttatacaatcttccaacaagttaaaactggcgaatttagacaacg
attcagtcataaaataaaggccttacgcatctcgagcgaaccagtacgagtgaaaattataacttcaaag
tctgcccggcacgtgacttagagtctcccgcgagaccggcgctccggagttctggaatgcagaagaagaa
gaagcattgtcacctaccgtgacgtcataaacgagtgtcttaaatagcgcctgcatcgagccatgcattc
tataacatcgctagccagccacgcatggtaaagttatcaagcgatagccagtcaatgatatatagtagcc
aatgtctgttataaccaggcatggtcacatagcgtgttttaatacaagtagatttacatggtatatagtt
agcggcgaactaatgttttaaactgcagcaaattgcagacattaggtcttgcaaaagtagcctacgagta
ttacattagccattcggtgatcaagttattttacacagaattcatacttaatgtttagtttgggagttcg
taataatatgttaattgcaattaaactgcaagccttatcgttgcacgaagtatattatataccagacttt
actacatatagaatcagaaagaatacagttacgaatcgccgacgaaacaagcttctaatattaaatcgtg
aaacattataaaagtgaatggattaaacttatcacgcttaaattatatcaagtcttcagggtatatactt
ttcgccggttctaaattatttttcgcaagacttgtttttatacaaaatcttaacctaaacgaatgtgca
gttataggtttaatattatgtgttacttgcaattacagtgcaaggagaaccgttgcaaaattacaaccat
aagttgcaagatattaagattatgtattgacctcatacattttgtatttcagaaatctagccggtagttt
gacatatttatacg
```

Development • Supplementary information

```
>Ciona MRF promoter (-2604/-6 version)
gcaagctcctttggggtttggccactcggtcggcaagtaaaatatcatttaagtttttaaatgtcgttta
aatggcagcagccgtaacaggtggactgccacgtgtcgcccggtgtgatcgtgtttccatcccaagggtg
gtagtgcaggcatgccgacgctcatatgagccattcataataatttggcgcatgtaagaggaaaatgcac
cagtaagatggtgccagcacattgtaattaaaatgtggtgctacggagaagtgtggaaatcgttatagga
aataaaatcccttattcccgatcgtttttaccaactaacgacgttattttatatttgcgaagttacggtt
acataattatagtagggtgggggaagatggaacaactttagcacagaatatataaatatcctgatcgagt
tctaaacaattaacaatggtccatgggttgcgaagatacggttttataattctttgaatgttctttgttt
gctaccaaacgggacgagaaaagaaattaaagcatgaaccattttacttcaacctactactacagtatct
aagtaatttcttttcgaaacagatttaacttctttgcgatttcaattagcgaatcccagtttgaccgtta
aagctatagcttagagtacgtggtgctaaatttcgttgattcgcgcccctgaagcgagcaacttattaga
tagaaatcccagctggtaacaacatgtgttcatcgccctactgatgtctgtggttgccgaactctcttta
cgagactgagagccgacgtctcgataaacgaggttgtgtattatgatttatttaggaaataaaaagtaag
aaaataaaaatttaattaagtaaaatacaaacgtttacaaaaattatgaatttttttcaccaattttaa
ttttattcaagtatccgacgacgtttaagaactttaaaatccaaatcctttgctgcttcctagaattata
ctgtggggtaagatggaaaccgtaaatattctgtgtttacaaccaaataggacgataaaagagaatgaaa
cgtggacaatttgccctaacacattacacggctaaaaatattaggtttcctcctaaaatagcagcgacga
ccataaggttattcgaagatgaaaggccaaagtaacggatgcaagtttttgcaagacaatgctgtaattg
ttacgtcacagaggaattccacaacttattccagagcagacgagcgtcgcgtttactgtttgctaaaaat
ggcgactcgaggtgacaattacaattcgcgctgcgaatgacgtaataatgctattcgctgtgatgaaata
atagaagtggaaattcgaacaataccaacccacgatgaagcatatgtagcgaatccggaattaaacctct
agacttgcttgggaaatgcatggcgtatatgagtgtattacgtgatcgttaaagcgtcttatacagtcgc
attagaagctattttaagaaaatccacggcccctcatgcaaatatagctgtgatctaataggttaggtta
gtttacattaagtttaataatgtcgatcacttattgcatgtttcatgatcttgcaaatatgatgacgttg
tatattgaggttgctgctttcagttttatacaatcttccaacaagttaaaactggcgaatttagacaacg
attcagtcataaaataaaggccttacgcatctcgagcgaaccagtacgagtgaaaattataacttcaaag
tctgcccggcacgtgacttagagtctcccgcgagaccggcgctccggagttctggaatgcagaagaagaa
gaagcattgtcacctaccgtgacgtcataaacgagtgtcttaaatagcgcctgcatcgagccatgcattc
tataacatcgctagccagccacgcatggtaaagttatcaagcgatagccagtcaatgatatatagtagcc
aatgtctgttataaccaggcatggtcacatagcgtgttttaatacaagtagatttacatggtatatagtt
agcggcgaactaatgttttaaactgcagcaaattgcagacattaggtcttgcaaaagtagcctacgagta
ttacattagccattcggtgatcaagttattttacacagaattcatacttaatgtttagtttgggagttcg
taataatatgttaattgcaattaaactgcaagccttatcgttgcacgaagtatattatataccagacttt
actacatatagaatcagaaagaatacagttacgaatcgccgacgaaacaagcttctaatattaaatcgtg
aaacattataaaagtgaatggattaaacttatcacgcttaaattatatcaagtcttcagggtatatactt
ttcgccggttctaaattatttttcgcaagacttgtttttatacaaaatcttaacctaaacgaatgtgca
gttataggtttaatattatgtgttacttgcaattacagtgcaaggagaaccgttgcaaaattacaaccat
aagttgcaagatattaagattatgtattgacctcatacattttgtatttcagaaatctagccggtagttt
gacatattt

>Ciona Mesp promoter (-1914/-1) from Davidson et al. 2005
cggttcaacgtgacgtcccatgccgatatcgtaaccatccggaacctctgatgctttttcaatatcatct
tttttgaaatccttcatttccgtttcatcgtcatttttgaaagccgggttctcatactcgtttttggtgc
tgtaaccgttttctgacatttttatctcatccaagtgcgaaccacttcaaagatggatagataccagatt
ttattacaaacaattagcaattcacgaaagttaaaaaaacgacataaaactataaaataaaattatactt
attaaatatgaagaaaaatatgcattttttaatcattctattgcaacaaatcggtatttttcgtattctct
tatacgaagattgcatacaagcttaacgtttcatctgtttccattggaataaaatagaaaacgtcccacc
gcctgccctaacttttataagcttcacaaattatcaaagtttaccgttgaactcctgataaatgattat
tcagctattttaaacccaacttcgtttataagtgatgccgtctccttcttcgtatgaccccgctctacta
aatacatgtttgcacactgatcctaatgaagaccgcacctgaacctttacatagccgatgcattcacata
taatgttctttgtgcaaccgaagtcgtatttgagcaaaccaaatatccaacttatataagtagaccttta
aaaatggacggtgaacaaactttgcgaagtcatcataattgaaggtttatttagctctcctattgtagg
```

Development • Supplementary information

gaaagatcctatggatgctccaaaacgataaacccgacttaccgaaaacgcggcggtcgttagaccatgc
ttattgataaaccacatacatacaaactttagcgcaaactcggctatatctttacaacattagacaaatt
ctctttcatgtattttcatgggattataaagtgtctattttaggtaacaagactttaataaaaatatcag
tgtcccatattgttggcggctttgtttgcgagtgtctctgtattgacaacgtttataattaacttggaag
taatgagtatacagcagcagacacagaagtttgtcgggggtatcctatgattgtacatcatgtgagcaaa
tatacttcaactgctagtgaataatatttgatatacgcacaagtcgtacaaatacacgaccacaaataag
tcatacttgtcgaagtttgtattcccctgttttcgatcaatcttatcagccacaaaaaatggaaaaattc
caaaaacgtagacacccacaaagtaacatacatggtaactcgtaagctaatacagagcacccgtgtgaaa
acgactgtcgttttccggccacgcgagaataaagtaagttacattgattcattcactgacagtttcccac
caaaactgaagtattaatcaattatgaatcggcagtgctttcagtgggaagatttcggaaaatgtgggtc
taatgtcacagtaaactttgtgtttgttttaaaaacaatcatcgttaaatcaacatcgcaattctgtcaa
tcactccacaataaactatatggctaatggaaacaggctgtttgtttgtatatcataaaatacttgcgtc
gtattatctgaccaaacaaaagcgttacaggaacggtccagcttcaaaatttgctgatgcatcagagttt
acatttgaaatgtgattaattacgaaaatccagcgaatagaattgtcacaacaagtcattagcgacggat
atttcgcctttgaaacttaaaggcgataatgactttgcccgtttcatgcggcgataaacgaactaattag
acacctcctacagatataatggtaattcagaatcgtgtggttatgtaattcgcaaaaacattttaacaaa
acaggttgatttgaaacttgtatt

>nls::Cas9::nls from Stolfi et al. 2014 START STOP
ATGGCTAGCCCCAAAAAGAAGAGGAAAGTGGACAAGAAGTATTCTATCGGACTGGACATCGGGACTAATA
GCGTCGGGTGGGCCGTGATCACTGACGAGTACAAGGTGCCCTCTAAGAAGTTCAAGGTGCTCGGGAACAC
CGACCGGCATTCCATCAAGAAAAATCTGATCGGAGCTCTCCTCTTTGATTCAGGGGAGACCGCTGAAGCA
ACCCGCCTCAAGCGGACTGCTAGACGGCGGTACACCAGGAGGAAGAACCGGATTTGTTACCTTCAAGAGA
TATTCTCCAACGAAATGGCAAAGGTCGACGACAGCTTCTTCCATAGGCTGGAAGAATCATTCCTCGTGGA
AGAGGATAAGAAGCATGAACGGCATCCCATCTTCGGTAATATCGTCGACGAGGTGGCCTATCACGAGAAA
TACCCAACCATCTACCATCTTCGCAAAAAGCTGGTGGACTCAACCGACAAGGCAGACCTCCGGCTTATCT
ACCTGGCCCTGGCCCACATGATCAAGTTCAGAGGCCACTTCCTGATCGAGGGCGACCTCAATCCTGACAA
TAGCGATGTGGATAAACTGTTCATCCAGCTGGTGCAGACTTACAACCAGCTCTTTGAAGAGAACCCCATC
AATGCAAGCGGAGTCGATGCCAAGGCCATTCTGTCAGCCCGGCTGTCAAAGAGCCGCGGACTTGAGAATC
TTATCGCTCAGCTGCCGGGTGAAAAGAAAAATGGACTGTTCGGGAACCTGATTGCTCTTTCACTTGGGCT
GACTCCCAATTTCAAGTCTAATTTCGACCTGGCAGAGGATGCCAAGCTGCAACTGTCCAAGGACACCTAT
GATGACGATCTCGACAACCTCCTGGCCCAGATCGGTGACCAATACGCCGACCTTTTCCTTGCTGCTAAGA
ATCTTTCTGACGCCATCCTGCTGTCTGACATTCTCCGCGTGAACACTGAAATCACCAAGGCCCCTCTTTC
AGCTTCAATGATTAAGCGGTATGATGAGCACCACCAGGACCTGACCCTGCTTAAGGCACTCGTCCGGCAG
CAGCTTCCGGAGAAGTACAAGGAAATCTTCTTTGACCAGTCAAAGAATGGATACGCCGGCTACATCGACG
GAGGTGCCTCCCAAGAGGAATTTTATAAGTTTATCAAACCTATCCTTGAGAAGATGGACGGCACCGAAGA
GCTCCTCGTGAAACTGAATCGGGAGGATCTGCTGCGGAAGCAGCGCACTTTCGACAATGGGAGCATTCCC
CACCAGATCCATCTTGGGGAGCTTCACGCCATCCTTCGGCGCCAAGAGGACTTCTACCCCTTTCTTAAGG
ACAACAGGGAGAAGATTGAGAAAATTCTCACTTTCCGCATCCCCTACTACGTGGGACCCCTCGCCAGAGG
AAATAGCCGGTTTGCTTGGATGACCAGAAAGTCAGAAGAAACTATCACTCCCTGGAACTTCGAAGAGGTG
GTGGACAAGGGAGCCAGCGCTCAGTCATTCATCGAACGGATGACTAACTTCGATAAGAACCTCCCCAATG
AGAAGGTCCTGCCGAAACATTCCCTGCTCTACGAGTACTTTACCGTGTACAACGAGCTGACCAAGGTGAA
ATATGTCACCGAAGGGATGAGGAAGCCCGCATTCCTGTCAGGCGAACAAAAGAAGGCAATTGTGGACCTT
CTGTTCAAGACCAATAGAAAGGTGACCGTGAAGCAGCTGAAGGAGGACTATTTCAAGAAAATTGAATGCT
TCGACTCTGTGGAGATTAGCGGGGTCGAAGATCGGTTCAACGCAAGCCTGGGTACCTACCATGATCTGCT
TAAGATCATCAAGGACAAGGATTTTCTGGACAATGAGGAGAACGAGGACATCCTTGAGGACATTGTCCTG
ACTCTCACTCTGTTCGAGGACCGGGAAATGATCGAGGAGAGGCTTAAGACCTACGCCCATCTGTTCGACG
ATAAAGTGATGAAGCAACTTAAACGGAGAAGATATACCGGATGGGGACGCCTTAGCCGCAAACTCATCAA
CGGAATCCGGGACAAACAGAGCGGAAAGACCATTCTTGATTTCCTTAAGAGCGACGGATTCGCTAATCGC
AACTTCATGCAACTTATCCATGATGATTCCCTGACCTTTAAGGAGGACATCCAGAAGGCCCAAGTGTCTG
GACAAGGTGACTCACTGCACGAGCATATCGCAAATCTGGCTGGTTCACCCGCTATTAAGAAGGGTATTCT
CCAGACCGTGAAAGTCGTGGACGAGCTGGTCAAGGTGATGGGTCGCCATAAACCAGAGAACATTGTCATC

Development • Supplementary information

GAGATGGCCAGGGAAAACCAGACTACCCAGAAGGGACAGAAGAACAGCAGGGAGCGGATGAAAAGAATTG
AGGAAGGGATTAAGGAGCTCGGGTCACAGATCCTTAAAGAGCACCCGGTGGAAAACACCCAGCTTCAGAA
TGAGAAGCTCTATCTGTACTACCTTCAAAATGGACGCGATATGTATGTGGACCAAGAGCTTGATATCAAC
AGGCTCTCAGACTACGACGTGGACCACATCGTCCCTCAGAGCTTCCTCAAAGACGACTCAATTGACAATA
AGGTGCTGACTCGCTCAGACAAGAACCGGGGAAAGTCAGATAACGTGCCCTCAGAGGAAGTCGTGAAAAA
GATGAAGAACTATTGGCGCCAGCTTCTGAACGCAAAGCTGATCACTCAGCGGAAGTTCGACAATCTCACT
AAGGCTGAGAGGGGCGGACTGAGCGAACTGGACAAAGCAGGATTCATTAAACGGCAACTTGTGGAGACTC
GGCAGATTACTAAACATGTCGCCCAAATCCTTGACTCACGCATGAATACCAAGTACGACGAAAACGACAA
ACTTATCCGCGAGGTGAAGGTGATTACCCTGAAGTCCAAGCTGGTCAGCGATTTCAGAAAGGACTTTCAA
TTCTACAAAGTGCGGGAGATCAATAACTATCATCATGCTCATGACGCATATCTGAATGCCGTGGTGGGAA
CCGCCCTGATCAAGAAGTACCCAAAGCTGGAAAGCGAGTTCGTGTACGGAGACTACAAGGTCTACGACGT
GCGCAAGATGATTGCCAAATCTGAGCAGGAGATCGGAAAGGCCACCGCAAAGTACTTCTTCTACAGCAAC
ATCATGAATTTCTTCAAGACCGAAATCACCCTTGCAAACGGTGAGATCCGGAAGAGGCCGCTCATCGAGA
CTAATGGGGAGACTGGCGAAATCGTGTGGGACAAGGGCAGAGATTTCGCTACCGTGCGCAAAGTGCTTTC
TATGCCTCAAGTGAACATCGTGAAGAAAACCGAGGTGCAAACCGGAGGCTTTTCTAAGGAATCAATCCTC
CCCAAGCGCAACTCCGACAAGCTCATTGCAAGGAAGAAGGATTGGGACCCTAAGAAGTACGGCGGATTCG
ATTCACCAACTGTGGCTTATTCTGTCCTGGTCGTGGCTAAGGTGGAAAAAGGAAAGTCTAAGAAGCTCAA
GAGCGTGAAGGAACTGCTGGGTATCACCATTATGGAGCGCAGCTCCTTCGAGAAGAACCCAATTGACTTT
CTCGAAGCCAAAGGTTACAAGGAAGTCAAGAAGGACCTTATCATCAAGCTCCCAAAGTATAGCCTGTTCG
AACTGGAGAATGGGCGGAAGCGGATGCTCGCCTCCGCTGGCGAACTTCAGAAGGGTAATGAGCTGGCTCT
CCCCTCCAAGTACGTGAATTTCCTCTACCTTGCAAGCCATTACGAGAAGCTGAAGGGGAGCCCCGAGGAC
AACGAGCAAAAGCAACTGTTTGTGGAGCAGCATAAGCATTATCTGGACGAGATCATTGAGCAGATTTCCG
AGTTTTCTAAACGCGTCATTCTCGCTGATGCCAACCTCGATAAAGTCCTTAGCGCATACAATAAGCACAG
AGACAAACCAATTCGGGAGCAGGCTGAGAATATCATCCACCTGTTCACCCTCACCAATCTTGGTGCCCCT
GCCGCATTCAAGTACTTCGACACCACCATCGACCGGAAACGCTATACCTCCACCAAAGAAGTGCTGGACG
CCACCCTCATCCACCAGAGCATCACCGGACTTTACGAAACTCGGATTGACCTCTCACAGCTCGGAGGGGA
TGAGGGAGCTCCCAAGAAAAAGCGCAAGGTAGGTTAATGA

>Ciona U6 promoter from Nishiyama and Fujiwara 2008
tggcgggtgtattaaaccactaaacaaacaattgccccaagctctcttcacaattataaacactacaaat
gtttggacaagagattagcgtggctgtgacgagtaatctcaaaggcttggtgtaattgatattttataag
aagcagattaaacttcaatacagttaacacctcatttacaaaaaattggctgccaaaatcgctaattaac
acatatttaaaacaatttctacagatatacacagtatagtatgattactaactgcataataaacaaacat
atccaacagacactcactaatctgccataacaagcttcaaaaacttaaactcgaaattttagtgaatctt
tttttttaatgaagattttatttaaaaagttaaaaatattacagttcaggtataggtttacacctaatct
ttaataatccgaactaaattttaactatttagaaactttttcaaccaaagtttaaaaaaatagattcttc
gcacgctaaaactatcatttacacaaaaaaatgcaacaaaatgcagaaaaaaattacattagagtttagg
ttagttacctgctaatcattataaactaacttccggcataatattcatctaaaattagcaataatcacgt
tttacgctaaaatttgtgtaaaactaaacttcgtcctttgtcaaggagaaaatttgactcaaaagctgcg
cgcgcaggggagatccccaagcgagtgtttgttacatcataatcatgtggaaaaatcccctaataagtaa
aaatacatatttttttaattttggggcaaataaaccgctttttatgtctaaaaacgccaaaaatggatcg
cgcgagcccaaaaacgcacaaataacgtacagacagtgtctctgcgtacacagacggtatttcccctta
aattgagaactagacttaagcacgcttataagtctggaaggcatccgatggtatagat

>Ciona Ebf promoter (-2631/+15+STOP) START from Stolfi and Levine 2011
attcttccgggaaataagagcggcagcgactttattcgaatttcaaatttccatgatattcgatacccca
cactccaccctataacggtgacctggtttaggaaatatgtgacgttgcttcacgacaattttagcagcta
tataagttaccattgcaaaaagatcagtctttttatttatcacaatttttaccagatttttaaatatttt
ttagaaataacttaaattttgatcaaactgggttaaaattgcaaattttacatttttgtgattaatatta
atctttttagtcgccatggcttaaaaatgtcatttgtttagtccgctgtattcaattttaccgtttgata
aatcaaacggttaagtttcgcataaaaccgatttctcatatggatattactccgcgcgagttctaacaac
gcgttctcttcgctggccaagatcgatactccgtgacgtcacaatcacatgcgcgcgcgtctgaaatatg

Development • Supplementary information

gtcgacagagttgcaacacgcaacccttttgtttgggttaatttccctctttgtttttaagtccgatcg
cgtcgccacatctgttcgacgaatcttcgttctgtcttagcaactttgcggctctgtccattgtgaagtc
gtaaacgaggcattgtcgtcactgctgtctcgcctacgtcacaaagcagtgcacagtgacgtcacggaga
cgaggtcgcgtgccttcgagttggaaaattcaaaccattgcacttttgccgaatgttaattttacgac
gggagaagttaggaaaagcataaaataacaaataaataaccaataaaaaacgtaaaaacaaacaagagaa
cggtattcaaaattagaatttcgaaaaaaatgttaaaaaataatacttagagtcgctgaacaatttaagc
cgacaaaggccacaaaactgcctaaaatttataaaaaaaaatgtaaaattattttgtttttttaaacta
cagttatcacctttaaaacaaacaaattagcaaacgttgtaattacttcacaactttcttgcgacgctaa
aaggcggcgaattttattgctattgtgacgtcacaagcgctctcgtcacgcccggatacgattagaacaa
cgaaggattgtttgtttttaattatttctctgtttaatcatttgatttagcgcggcacaaattttgtttt
atataaaatgtatctatttatccattttatttctgtgcgttgttgtactatttttttgaaaaatgtttgtt
aacctttagaaaatcgcgaaaccaacgaaattatttctaaaggctgtacaattctttttcgttaggttac
gtgtttaagtataggaccaagttttaaatggcggacagagtttcgtattttgatatcttgaattattttg
aaatcttgaaaaaaaataattgtacgctttacatagaataactaaccaaaatatctgaagcaaaaattac
gtacaatttttaaaatgtaatttactttctagcttttaatttttgtgcttttctcaatatgtgtgccat
attttaaaaacgtaaatttgcctgttgttaagcggaggaaaaagtaatctgcgtgaatgcgaaaaacacg
attttggaatcagcgccggcaatggtgtttgtaaataggggtggcatacgcgtttcgtagcgaaagagag
aatgaggcgaaagtcgacagatgcacgctccgatttatgagacaggaaccagtcgcgagggcacggagga
aaaagaaccttactccaagacatgcgccgcctttttttctttctgtcctagtcaggaatactagagtatag
aaggccacgcgtcgtggagtttaaaaccagcaagcgagtgtctcaacggacacatcaatacagagacttc
tctcagtggacaactcggacgattcgccactaacttggtggattcgtcccggacgacccatgggccgggt
cccagcgcgctagttggccaccatacagtgtagaatcagctagatcgtctctgcggatttcgcaaataga
ttgagttggagataggttcccgaccgggatttcgactaatttgcaatgttagttattaatcaaggtgaca
gtcaggagttaaagttaatttaccttttgaaagggcaaaaagattttcgaagtaaattgattccgttaat
tgtaactcttaaacgcaaaccgataaacgacgccattttgcttttcattgagaaactaaccatttaggca
ttctataattaaaattaaatgtttttaaaatctgtaactatctgtaataaactagaattatttatttcag
tttaaattttatttaaaacaataattttattatttcctttattcatcctattgatattggtatagagaa
aacgatgttttatttccaaaaaatttttgtttcataaaagtcattttgttcaatttaaaaaaatacctta
ttctaaaaaatgcccaaaacaagttctttatttcttaaaaactatcctaaataaaaaaaaactccacgtt
ttaataaaacctataaaattaaaaactataaaaagatgacttatttttttaccctaacgtgattttcac
cagataccttaagtgttattttatttgtaagtaatatccaaATGGCAACAATCGCGTAA

>Ciona Ebf START STOP
ATGGCAACAATGGCGGGACCTCAGTTATCGGGGCCAGCAGTGAGAGGATGGATGCAAACAACGTTAGTGG
AACCGATGCCAAACGGCAATGTTGGTCTACATCGGGCACATTTCGAAAAGCAGCCGCCAAACAACCTTAG
AAAAAGCAACTTTTTCCACTTCGTGCTTGCCCTGTACGACAGACAAGGGCAGCCGGTGGAAATTGAGCGA
AGTGCTTTCGTAGGATTTGTGGAAAACGAAACCGAGATCGCCGGCGAAAAAACAAACAACGGGATCCAGT
ACAGACTCCAATTGCTTTACCATAGCGGCGTACGGACAGAACAAGATGTATTCGTCCGTCTTATCGACTC
TGCAACCAAACAGTCAATTACGTACGAAGGACAGGATAAAAACCCAGAAATGCGACGGGTTTTGCTCACT
CATGAGATCATGTGCAGCCGCTGCTGTGACAAGAAGAGTTGCGGGAACAGAAACGAAACTCCATCCGACC
CAGTGGTGATTGACAGATACTTCTTAAAATTCTTTCTCAAGTGCAACCAAAACTGTTTAAAGAATGCTGG
AAATCCAAGAGACATGCGAAGATTTCAGGTTGTGGTCTCAACAACTGTGCATGTTGACGGTCATGTTTTA
GCTGTGTCTGACAACATGTTCGTGCACAACAACTCGAAACATGGACGTCGAGCGAGAAGGGTCGACCCAT
CAGAAGCTTCGCCAACTATCAAAGCAATCAACCCAGCTGAGGGTTGGACAACAGGTGGAGCAACAGTTGT
TATAGTTGGAGAAAATTTCTTTGACGGGCTTCAAGTTGTGTTTGGTTCAATGGTTGTATGGAGTGAGTTG
ATCACCCAACACGCAATCAGAGTACAGACTCCACCGCGCCATCTGCCGGGAGTGGTAGAAGTGACTCTGT
CTTACAAAAATAAACAATTCTGCAGTGGTGCTCCAGGACGCTTTGTATACACAGCGCTCAACGAACCGAC
ACTTGATTATGGATTCCAGCGATTACTGAAGACTGTGCCAAGACACCCAGGCGACCCTGAGAGGCTACCA
AAGGAGATTATTCTAAAACGAGCTGCTGACGTCATGGAAGCGGTGATCAGTCGGCAGTACGCCCCGCCAA
GCCAAATGCCCCCGTCAGCCGGGATCACGCCTCCGGCGCCCCATCTAGCCGCGGCACCGTGCGCACCACC
TGGCAGTTTCGTTCCGCAATCTGCTAGCGCTGCCATGGCCGTGGCAATGAACGGTTACGCTGCTGCAGCG
GTTTCTTCACAATTTGGCGGAACACCCGATCGGTTCGACACCGGAAGTGATTCAGGTTACTCACGAGGTA

Development • Supplementary information

ACAGTGTTTCTCCCCGCAACGGATATTCCCCCCAGACGACACCGCATAGCTTGAACAGTGGCTCAATTGG
TAGCATGGTGGGGCTCACAACTGTAGGCGCAGTTCCGGCCCCAGCCCCCTACCACTGCGCACCATCCTTT
AACAGTTATTCCAGCGCTTCAGGTCCCACATTCACGAACATGAACAACACGTCTCCTGGCCTTTTTTCTG
GATCTGGGATAATTCCACCTTCGCCCCACAACGGTATGAACCCACTCCCGTCTTCGGGGACCACACCGGG
AATTTTTAGTTTTTCACCTGCAAACATGATATCAGCCGCAAAGCAGAAGAGCGCATTTGCCCCTGTACAT
CGTCCACACAACTCGCCCAGTCCTCTAGCGCCATCTAACGGAAATATCGCACTAAACGGCTATAGCTAG

>Human EBF3 (codon optimized for C. elegans) START STOP
ATGTTCGGAATCCAAGAAAACATACCAAGAGGCGGTACTACTATGAAAGAAGAACCACTTGGATCAGGAA
TGAATCCAGTTAGATCATGGATGCATACTGCTGGAGTCGTTGATGCGAATACAGCTGCGCAATCTGGAGT
TGGACTTGCTAGAGCACATTTTGAGAAACAACCACCAAGTAATTTACGAAAGTCAAACTTCTTTCATTTT
GTCTTGGCCCTTTATGACAGACAAGGTCAACCTGTAGAAATCGAGAGAACAGCGTTCGTTGATTTCGTTG
AAAAGGAAAAGGAACCTAATAATGAGAAGACTAATAATGGTATCCATTACAAGCTTCAATTGTTATATAG
TAATGGGGTTAGGACCGAGCAGGACCTTTACGTCAGACTGATTGACAGCATGACTAAGCAAGCAATTGTT
TATGAAGGGCAAGATAAGAATCCTGAAATGTGTAGGGTTTTGCTTACGCATGAAATTATGTGTTCGCGTT
GTTGCGATAAGAAGTCTTGCGGAAACCGAAATGAGACCCCTAGTGATCCAGTCATTATAGATAGGTTCTT
CCTGAAATTCTTTCTTAAATGTAACCAAAATTGCCTTAAGAACGCCGGAAATCCACGTGACATGCGCCGG
TTTCAAGTAGTAGTCTCTACCACCGTAAATGTTGATGGACATGTATTGGCTGTTTCTGATAATATGTTCG
TTCATAATAACTCAAAGCATGGACGACGAGCACGTCGTTTGGATCCAAGCGAGGGAACAGCTCCATCATA
CCTTGAGAACGCAACACCATGTATTAAAGCTATTTCTCCGTCCGAGGGATGGACGACTGGCGGCGCAACA
GTGATTATTATCGGAGATAATTTCTTCGATGGCCTTCAGGTGGTTTTCGGTACGATGCTTGTTTGGAGTG
AATTGATCACACCTCACGCGATTCGGGTTCAAACGCCACCTCGTCATATCCCGGGAGTTGTTGAGGTAAC
GCTTTCTTATAAGTCGAAACAATTTTGTAAGGGAGCACCGGGCAGGTTCGTATATACTGCATTAAACGAG
CCGACAATTGACTATGGATTCCAACGTCTGCAAAAGGTTATACCGCGTCACCCAGGAGACCCAGAGCGCT
TGCCAAAAGAAGTTCTTTTGAAAAGGGCCGCTGATTTGGTAGAGGCATTGTATGGTATGCCACATAATAA
TCAAGAAATTATATTAAAGAGAGCCGCTGATATTGCAGAGGCTCTCTATTCTGTCCCAAGGAACCATAAT
CAAATACCAACACTTGGAAATAATCCAGCCCATACCGGAATGATGGGAGTTAATAGCTTTTCGTCACAAC
TGGCTGTGAATGTCTCGGAAACTTCCCAGGCTAATGATCAGGTGGGGTATTCACGGAACACCTCTTCGGT
CTCACCAAGAGGATATGTTCCGTCATCCACGCCACAACAATCAAACTATAATACTGTTTCAACCAGTATG
AACGGGTACGGGAGCGGAGCGATGGCAAGCCTTGGAGTACCGGGATCACCGGGTTTCCTGAACGGTTCAA
GTGCCAATTCACCATATGGAATCGTTCCCTCAAGTCCTACAATGGCTGCTTCATCTGTAACGCTTCCATC
CAATTGCTCATCCACTCATGGAATCTTTTCGTTTTCTCCGGCTAACGTAATTTCAGCGGTCAAGCAAAAG
TCAGCATTTGCACCAGTTGTTAGACCACAGGCTTCCCCACCACCATCATGTACGTCTGCGAATGGAAACG
GTTTGCAGGGAAGCCTTCTCGGAGCGGAAGATGTGGCGGCTGAGAAAACGAATTGGCCTTTCTGCGAGGT
TGGTGGGATTTTCCATTTCGACGAGCTTATGCTAAAGAAGGGCACAGGGAAATTGTGCTTAGGTTGGTAA

>Ciona MRF (transcript variant 1, with sgRNA mismatches) START STOP
ATGACGTGTATCTCTCTAGAGGAGCTCGACCTCTCTTCAATATTCTCCAACAGCAGCAGCTATTTCACCA
GTTACGCCACAAACCCCATTATGACGTCACAAAAACGGACGCCTTCACGACTGAAGCGAGCAAGCAGCGA
CGTTTTGTTGTCAGATACAGCGGTTTCGCCGGTAAGTCGAGAAGTTTCGGGGGTTTTATCGGAACTGGAC
GAACTGAAGAGATGtGTcGAGGGAAATTATATCGGAATCGATGCGGAAAAGAGCGATATCTCGATACTTG
AGGAACTTAGTAATATGGCGTCCGGTTGTAGCGATTCAGACGTCGCTTACTCGTCCCCGGATTCGAGGTA
CGGGTCAACTGGTAACCTTACCAGCAAGACAAGTTTCGGATCGGGAATGGGTTTCCGTGACGCTGGGATT
GGAGGGCCAGTTTCCCGTGGAAGTTCGCTACGAAACCCCGATATTCGGGGCAAACGTAACTCCATTGAGG
TGAAGCAAGAAAACGAACAAAACCCGATCGAGTTTGTTTTAGAAAGCTTCCTCAACTCAAACGAAACCTC
CAAACCCAGGATCCATGAGAGCAGAGTTGAAGACATCCAGTTCACAGGACCCCCTATTAGCAGCATTGAG
CCGACAATCACATTTGCATCAACAGAGGAGAATCACAACGACACAGTGAAAGCTATGATGCAATACTTAA
CCGAGACCAACCAAATGTGTCAAGAAAACAGTGTTCCCGAGCAAATAATCTTCAGCGACTTAAACAGTGT
TTCCGCATCCGATTCTCTTCCAAGCGTTGAAGAATTGCTTCAAATTCCGAACGAAAAAAGCCGCAGTTTT
AAACCTGACCACACTGCGAAGCACCAGCCGACGCTGAACCGCCCAAAACATTTCCACAACCAAGTAGTCA
ACCAGCCACATGTACCAATCCTAAACCCAGAGTTACAAAGCTTTGAAAACTTCAGCCCCGTGATAAACAA
CAGTCTCATCACTCCAACCAACACGTTCACATCCAACCGCATGATGGAGCTATCACCACTATCAGATCTT

Development • Supplementary information

CATAGTTTATCGCAGGATGAAGACATGGACACAAAGATGTCGCATTATCACCATACAAGCCACCCAAACG
GCCATCAATGTTTAGTATGGGCATGCAAAGCGTGTAAACGTAAAACTGGCCACACGAtCGtCGGAGGGC
GGCAACACTACGAGAGAGACGACGCCTTAAACGTGTCAACCAAGCGTACGACGCCCTTAAGCGTTGCGCA
TGCGCAAACCCGAACCAGAGACTTCCGAAAGTTGAGATTCTTCGCAACGCGATAACCTACATATACAATC
TACAACATATGTTGTATGGCGACCAACAGTCTGATGCAAAATCACCAGAAACCAAACCAGAAACGACTTT
AAGCTTGGGCGAAACTTTCGTTAGCAAAACCGAAGTTGATTCGCCGTTTTATCAGTCCGATGACGTCAGA
CTTACGTCATCAAGGACGTCAAGCCCGGTTGAGTCCCTTCTTGAGTCCACGTCATCGTCCTTCATAATGT
CGGATTTGGGCGACGAAAATACTCAACCTCAGGTACTTTAA

>Ciona MRF (transcript variant 2, with sgRNA mismatches) START STOP
ATGACGTGTATCTCTCTAGAGGAGCTCGACCTCTCTTCAATATTCTCCAACAGCAGCAGCTATTTCACCA
GTTACGCCACAAACCCCATTATGACGTCACAAAAACGGACGCCTTCACGACTGAAGCGAGCAAGCAGCGA
CGTTTTGTTGTCAGATACAGCGGTTTCGCCGGTAAGTCGAGAAGTTTCGGGGGTTTTATCGGAACTGGAC
GAACTGAAGAGATGtGTcGAGGGAAATTATATCGGAATCGATGCGGAAAAGAGCGATATCTCGATACTTG
AGGAACTTAGTAATATGGCGTCCGGTTGTAGCGATTCAGACGTCGCTTACTCGTCCCCGGATTCGAGGTA
CGGGTCAACTGGTAACCTTACCAGCAAGACAAGTTTCGGATCGGGAATGGGTTTCCGTGACGCTGGGATT
GGAGGGCCAGTTTCCCGTGGAAGTTCGCTACGAAACCCCGATATTCGGGGCAAACGTAACTCCATTGAGG
TGAAGCAAGAAAACGAACAAAACCCGATCGAGTTTGTTTTAGAAAGCTTCCTCAACTCAAACGAAACCTC
CAAACCCAGGATCCATGAGAGCAGAGTTGAAGACATCCAGTTCACAGGACCCCCTATTAGCAGCATTGAG
CCGACAATCACATTTGCATCAACAGAGGAGAATCACAACGACACAGTGAAAGCTATGATGCAATACTTAA
CCGAGACCAACCAAATGTGTCAAGAAAACAGTGTTCCCGAGCAAATAATCTTCAGCGACTTAAACAGTGT
TTCCGCATCCGATTCTCTTCCAAGCGTTGAAGAATTGCTTCAAATTCCGAACGAAAAAAGCCGCAGTTTT
AAACCTGACCACACTGCGAAGCACCAGCCGACGCTGAACCGCCCAAAACATTTCCACAACCAAGTAGTCA
ACCAGCCACATGTACCAATCCTAAACCCAGAGTTACAAAGCTTTGAAAACTTCAGCCCCGTGATAAACAA
CAGTCTCATCACTCCAACCAACACGTTCACATCCAACCGCATGATGGAGCTATCACCACTATCAGATCTT
CATAGTTTATCGCAGGATGAAGACATGGACACAAAGATGTCGCATTATCACCATACAAGCCACCCAAACG
GCCATCAATGTTTAGTATGGGCATGCAAAGCGTGTAAACGTAAAACTGGCCACACGAtCGtCGGAGGGC
GGCAACACTACGAGAGAGACGACGCCTTAAACGTGTCAACCAAGCGTACGACGCCCTTAAGCGTTGCGCA
TGCGCAAACCCGAACCAGAGACTTCCGAAAGTTGAGATTCTTCGCAACGCGATAACCTACATATACAATC
TACAACATATGTTGTATGGCGACCAACAGTCTGATGCAAAATCACCAGAAACCAAACCAGAAACGACTTT
AAGCTTGGGCGAAACTTTCGTTAGCAAAACCGAAGTTGATTCGCCGTTTTATCAGTCCGATGACGTCAGA
CTTACGTCATCAAGGACGTCAAGCCCGGTTGAGTCCCTTCTTGAGTCCACGTCATCGTCCTTCATAATGT
CGGATTTGGGCGACGAAAATACTCAACCTCAGGATACGTTCCCGGTAAACTTGCTTACTGATGACGTCAC
CAGACCCTCGTCAACAACCCCTGACGTCATCGCTGTCGTAAACGAACCAACAACAACAACGGAAGATCGA
AACTCGAGCCCTGTGACGTCAGTGAGCAACAGCGACACCAAGGGAGCTTCTAGTTTGGTTTGTTTGACGT
CGATCGTGGAAAGGATCGATTAA

>Human MYOD1 (codon optimized for C. elegans) START STOP
ATGGAACTGTTATCACCGCCTCTTCGAGATGTCGATCTTACAGCACCAGATGGTTCCCTGTGTAGTTTCG
CAACTACAGATGACTTTTACGATGATCCATGCTTTGATTCTCCAGATCTTCGTTTCTTTGAGGATCTCGA
TCCAAGATTGATGCATGTCGGTGCTTTGTTAAAGCCTGAGGAACATTCACATTTTCCAGCAGCTGTCCAT
CCTGCTCCAGGAGCTCGCGAAGATGAACACGTACGGGCACCATCAGGACATCATCAAGCTGGTCGATGTC
TTCTTTGGGCTTGTAAAGCTTGTAAACGAAAGACAACGAATGCAGATCGTAGAAAAGCTGCTACAATGAG
AGAAAGAAGAAGACTTAGTAAGGTGAACGAAGCTTTCGAGACGCTTAAACGTTGTACTTCAAGCAACCCT
AATCAAAGGCTCCCAAAAGTTGAAATTTTAAGAAATGCAATTAGATACATAGAAGGATTGCAAGCCCTAC
TTCGTGATCAAGATGCTGCACCGCCGGGAGCAGCTGCAGCATTTTACGCCCCAGGACCATTGCCACCAGG
ACGAGGTGGAGAACATTATTCAGGGGATTCAGATGCTTCATCACCCCGAAGCAATTGTTCGGATGGAATG
ATGGATTATTCTGGTCCGCCCTCAGGGGCTAGAAGACGAAATTGTTATGAGGGTGCTTATTATAATGAAG
CCCCGTCTGAGCCTAGACCTGGCAAATCAGCAGCTGTCAGTTCATTGGATTGTTTAAGTAGTATTGTTGA
ACGAATTAGCACAGAATCGCCAGCCGCACCAGCTCTACTTTTAGCAGATGTCCCGAGTGAAAGTCCACCC
CGGAGACAGGAAGCAGCAGCTCCAAGTGAAGGTGAATCGAGTGGTGATCCAACACAATCCCCTGATGCTG
CACCACAATGTCCCGCTGGAGCTAATCCAAATCCAATTTATCAAGTTTTGTAG

Development • Supplementary information

>Ciona Mymk promoter (-508/-1), mEbf
tgctctggaaaatttaccaagggaaactccctcacgtggtaacatagaaatagtacaaaaaatgcttaaa
attgtttttcaccaaaactaacggtacacataccgttgcctaaactaggcaaaacacatattagattgca
tttaaactggtaaatgtcctaataatttaaaacttgcattaacaataaatttagttgccaataatataac
ttttaaattgcgtattttaagtatgtacatagttaagaatatttattcgggccataaatgtgtattttaa
agttgtttatatttagaccaaatactgatattttaataaacgttttgggtcgtgagaaatatcacattat
tgtgtccagctgaatcagacaacagctgaagaccacatgccaattAAAaagataatggtgcagcacgcgc
aaaacaaccgagagtaccgtagtacatttggtatgtcgcctttagagcattcattgcatagtgttaaacc
gtagattaaatcatagtt

>Ciona Mymk promoter (-508/-1), mMRF-136
tgctctggaaaatttaccaagggaaactccctcacgtggtaacatagaaatagtacaaaaaatgcttaaa
attgtttttcaccaaaactaacggtacacataccgttgcctaaactaggcaaaacacatattagattgca
tttaaactggtaaatgtcctaataatttaaaacttgcattaacaataaatttagttgccaataatataac
ttttaaattgcgtattttaagtatgtacatagttaagaatatttattcgggccataaatgtgtattttaa
agttgtttatatttagaccaaatactgatattttaataaacgttttgggtcgtgagaaatatcacattat
tgtgtccagctgaatcagacaaACgcGTaagaccacatgccaattcccaagataatggtgcagcacgcgc
aaaacaaccgagagtaccgtagtacatttggtatgtcgcctttagagcattcattgcatagtgttaaacc
gtagattaaatcatagtt

>Ciona Mymk promoter (-508/-1), mMRF-152
tgctctggaaaatttaccaagggaaactccctcacgtggtaacatagaaatagtacaaaaaatgcttaaa
attgtttttcaccaaaactaacggtacacataccgttgcctaaactaggcaaaacacatattagattgca
tttaaactggtaaatgtcctaataatttaaaacttgcattaacaataaatttagttgccaataatataac
ttttaaattgcgtattttaagtatgtacatagttaagaatatttattcgggccataaatgtgtattttaa
agttgtttatatttagaccaaatactgatattttaataaacgttttgggtcgtgagaaatatcacattat
tgtgtcACgcGTaatcagacaacagctgaagaccacatgccaattcccaagataatggtgcagcacgcgc
aaaacaaccgagagtaccgtagtacatttggtatgtcgcctttagagcattcattgcatagtgttaaacc
gtagattaaatcatagtt

> Ciona Mymk promoter (-508/-1), mHES
tgctctggaaaatttaccaagggaaactccctACcgGTgtaacatagaaatagtacaaaaaatgcttaaa
attgtttttcaccaaaactaacggtacacataccgttgcctaaactaggcaaaacacatattagattgca
tttaaactggtaaatgtcctaataatttaaaacttgcattaacaataaatttagttgccaataatataac
ttttaaattgcgtattttaagtatgtacatagttaagaatatttattcgggccataaatgtgtattttaa
agttgtttatatttagaccaaatactgatattttaataaacgttttgggtcgtgagaaatatcacattat
tgtgtccagctgaatcagacaacagctgaagaccacatgccaattcccaagataatggtgcagcacgcgc
aaaacaaccgagagtaccgtagtacatttggtatgtcgcctttagagcattcattgcatagtgttaaacc
gtagattaaatcatagtt

>Human MYMK -427/+3, based on Zhang et al. 2020 START
tctctactaaaaatacaaaaattagctgggtgtggtggcaggcacctgtcatcccacctattccagaggc
taaggcaggagaatctcttgaacctggaaggtggaggttgcagtgagccgagatcacaccactgcactcc
agcctgggtgacagggcgagactctgtctcagaaaaagaaaagaaaaatcagagacaaatgctggtcacg
tggcatgtcagctgttggccctctccgtgtgttactcgacacgtgcacgcacatccccgcctccgtcaag
ggcatttaaaccctcttgtgggtgctccccgcagctgccatcagagccctgcccaaagggagctggcctt
cccacttcgtgctcctgtgctggggacctgggacaccagcaccctccccaccccagccagtgctttcctc
ctggcccatg
>Chicken Mymk -499/+3, based on Luo et al. 2015
tgctcctattcctccctgcagagggaaagctcagctcctgaatttctatggggttgagtttcacccttcc
tgagcacccaggcacacgcgtgtataggtggcatttcacaccggataggatgctggctcagatttgacgc

tgcttccatgtctgttgctccccagcacctctcctccaggaataaaagccagattcagagctccgtgggt
gtgtgcaataaacgcgtgtgcatcacagccagcatgaagcgaaccacagccgtcaaaggacacaaacagc
cggggcattgcagggcacggagcatttgctgccccaagaattttgctatccaagaaggaatggagcacat
gggtggctcagctgtgccactgcctgcagctgttccctgcctgcagctctccgggcatttaaaatttaaa
tccttttctccacgtctgttttctcctccatctccagcagctcacacggacccaggagccgccggccctc
caggcaccc

>Ciona Mymk -508/-1 optEbf
tgctctggaaaatttaccaagggaaactccctcacgtggtaacatagaaatagtacaaaaaatgcttaaa
attgtttttcaccaaaactaacggtacacataccgttgcctaaactaggcaaaacacatattagattgca
tttaaactggtaaatgtcctaataatttaaaacttgcattaacaataaatttagttgccaataatataac
ttttaaattgcgtattttaagtatgtacatagttaagaatatttattcgggccataaatgtgtattttaa
agttgtttatatttagaccaaatactgatattttaataaacgttttgggtcgtgagaaatatcacattat
tgtgtccagctgaatcagacaacagctgaagaccacatgccaaCCcccaGgGGaatggtgcagcacgcgc
aaaacaaccgagagtaccgtagtacatttggtatgtcgcctttagagcattcattgcatagtgttaaacc
gtagattaaatcatagtt

>Ciona Mymk -508/-1 optMRF-136
tgctctggaaaatttaccaagggaaactccctcacgtggtaacatagaaatagtacaaaaaatgcttaaa
attgtttttcaccaaaactaacggtacacataccgttgcctaaactaggcaaaacacatattagattgca
tttaaactggtaaatgtcctaataatttaaaacttgcattaacaataaatttagttgccaataatataac
ttttaaattgcgtattttaagtatgtacatagttaagaatatttattcgggccataaatgtgtattttaa
agttgtttatatttagaccaaatactgatattttaataaacgttttgggtcgtgagaaatatcacattat
tgtgtccagctgaatcagacGGcagctgCCgaccacatgccaattcccaagataatggtgcagcacgcgc
aaaacaaccgagagtaccgtagtacatttggtatgtcgcctttagagcattcattgcatagtgttaaacc
gtagattaaatcatagtt

>Ciona Mymk -508/-1 optMRF-152
tgctctggaaaatttaccaagggaaactccctcacgtggtaacatagaaatagtacaaaaaatgcttaaa
attgtttttcaccaaaactaacggtacacataccgttgcctaaactaggcaaaacacatattagattgca
tttaaactggtaaatgtcctaataatttaaaacttgcattaacaataaatttagttgccaataatataac
ttttaaattgcgtattttaagtatgtacatagttaagaatatttattcgggccataaatgtgtattttaa
agttgtttatatttagaccaaatactgatattttaataaacgttttgggtcgtgagaaatatcacattat
tgtgGGcagctgCCtcagacaacagctgaagaccacatgccaattcccaagataatggtgcagcacgcgc
aaaacaaccgagagtaccgtagtacatttggtatgtcgcctttagagcattcattgcatagtgttaaacc
gtagattaaatcatagtt

>Ciona Mymk -508/-1 optMRF-152 + optMRF-136
tgctctggaaaatttaccaagggaaactccctcacgtggtaacatagaaatagtacaaaaaatgcttaaa
attgtttttcaccaaaactaacggtacacataccgttgcctaaactaggcaaaacacatattagattgca
tttaaactggtaaatgtcctaataatttaaaacttgcattaacaataaatttagttgccaataatataac
ttttaaattgcgtattttaagtatgtacatagttaagaatatttattcgggccataaatgtgtattttaa
agttgtttatatttagaccaaatactgatattttaataaacgttttgggtcgtgagaaatatcacattat
tgtgGGcagctgCCtcagacGGcagctgCCgaccacatgccaattcccaagataatggtgcagcacgcgc
aaaacaaccgagagtaccgtagtacatttggtatgtcgcctttagagcattcattgcatagtgttaaacc
gtagattaaatcatagtt

>Ciona Mymk -508/-1 optMRF-152 + optMRF-136 + mEbf
tgctctggaaaatttaccaagggaaactccctcacgtggtaacatagaaatagtacaaaaaatgcttaaa
attgtttttcaccaaaactaacggtacacataccgttgcctaaactaggcaaaacacatattagattgca
tttaaactggtaaatgtcctaataatttaaaacttgcattaacaataaatttagttgccaataatataac
ttttaaattgcgtattttaagtatgtacatagttaagaatatttattcgggccataaatgtgtattttaa

agttgtttatatttagaccaaatactgatattttaataaacgttttgggtcgtgagaaatatcacattat
tgtgGGcagctgCCtcagacGGcagctgCCgaccacatgccattAAAaagataatggtgcagcacgcgca
aaacaaccgagagtaccgtagtacatttggtatgtcgcctttagagcattcattgcatagtgttaaaccg
tagattaaatcatagtt

sgRNAs:

MRF.2 sgRNA **G**+(**N19**)
GGACGAACTGAAGAGATGCG

MRF.3 sgRNA **G**+(**N19**)
GTAGTGTTGCCGCCCTCCGT

Control sgRNA **G**+(**N19**) from Stolfi et al. 2014
GCTTTGCTACGATCTACATT

Electroporation mixes:

MRF CRISPR in ASM lineage
100 ug Mymk>Unc-76::GFP
40 ug U6>MRF.2
40 ug U6>MRF.3
40 ug Mesp>Cas9
40 ug Mesp>mScarlet

Negative control CRISPR in ASM lineage
100 ug Mymk>Unc-76::GFP
80 ug U6>Control
40 ug Mesp>Cas9
40 ug Mesp>mScarlet

MRF>Ebf to see ectopic Mymk reporter in tail muscles
50 ug MRF>Ebf
70 ug Mymk>Unc-76::GFP
10 ug MRF>H2B::mCherry

MRF>EBF3(human) to see ectopic Mymk reporter in tail muscles
50 ug MRF>EBF3(human)
70 ug Mymk>Unc-76::GFP
10 ug MRF>H2B::mCherry

Negative control to compare to Ebf/EBF3 overexpression
70 ug Mymk>Unc-76::GFP
10 ug MRF>H2B::mCherry

Ebf>MRF(Tv1) to see ectopic Mymk reporter in larval CNS
50 ug Ebf>MRF(Tv1)
70 ug Mymk>Unc-76::GFP
50 ug Ebf>CD4::mCherry

Development • Supplementary information

Ebf>MRF(Tv2) to see ectopic Mymk reporter in larval CNS
50 ug Ebf>MRF(Tv1)
70 ug Mymk>Unc-76::GFP
50 ug Ebf>CD4::mCherry

Ebf>MYOD1(human) to see ectopic Mymk reporter in larval CNS
50 ug Ebf>MYOD1(human)
70 ug Mymk>Unc-76::GFP
50 ug Ebf>CD4::mCherry

Negative control to compare to MRF/MYOD1 overexpression
50 ug Ebf>lacZ
70 ug Mymk>Unc-76::GFP
50 ug Ebf>CD4::mCherry

MRF>MRF(Tv1) to test effect of increased MRF dose on Mymk reporter
50 ug MRF>MRF(Tv1)
70 ug Mymk>Unc-76::GFP
10 ug MRF>H2B::mCherry

MRF>Ebf to see tail muscle cell shape at 24 hpf
50 ug MRF>Ebf
30 ug MRF>CD4::GFP
10 ug MRF>H2B::mCherry

Negative control to see tail muscle cell shape at 24 hpf
30 ug MRF>CD4::GFP
10 ug MRF>H2B::mCherry

MRF>Ebf for RNAseq
50 ug MRF>Ebf

Negative control for RNAseq
50 ug MRF>CD4::GFP

Wild type Mymk reporter scoring at 44 hpf
100 ug Mymk(wt)>Unc-76::GFP
100 ug Mymk(wt)>Unc-76::mCherry

Mymk (mEbf) reporter scoring at 44 hpf
100 ug Mymk(mEbf)>Unc-76::GFP
100 ug Mymk(wt)>Unc-76::mCherry

Mymk (mMRF-136) reporter scoring at 44 hpf
100 ug Mymk(mMRF-136)>Unc-76::GFP
100 ug Mymk(wt)>Unc-76::mCherry

Mymk (mMRF-136+mEbf) reporter scoring at 44 hpf
100 ug Mymk(mMRF-136+mEbf)>Unc-76::GFP
100 ug Mymk(wt)>Unc-76::mCherry

Mymk (mMRF-152) reporter scoring at 44 hpf

Development • Supplementary information

100 ug Mymk(mMRF-152)>Unc-76::GFP
100 ug Mymk(wt)>Unc-76::mCherry

Mymk (mHES) reporter scoring at 24 or 44 hpf
100 ug Mymk(mHES)>Unc-76::GFP

Wild type Mymk to compare to mHES reporter at 24 or 44 hpf
100 ug Mymk(wt)>Unc-76::GFP

Human MYMK -427/+3 reporter test
100 ug Human MYMK -427/+3>Unc-76::GFP

Chicken Mymk -499/-1 reporter test
100 ug Chicken Mymk -499/-1>Unc-76::GFP

Scoring wild type Mymk reporter expression in larval CNS
50 ug Ebf>lacZ
70 ug Mymk(wt)>Unc-76::GFP
50 ug Ebf>CD4::mCherry

Scoring effect of optimized Ebf site on expression in larval CNS
70 ug Mymk(optEbf)>Unc-76::GFP
50 ug Ebf>CD4::mCherry

Scoring wild type Mymk reporter expression in tail muscles
70 ug Mymk(wt)>Unc-76::GFP
10 ug MRF>H2B::mCherry

Scoring effect of optimized MRF-152 site on expression in tail muscles
70 ug Mymk(optMRF-152)>Unc-76::GFP
10 ug MRF>H2B::mCherry

Scoring effect of optimized MRF-136 site on expression in tail muscles
70 ug Mymk(optMRF-136)>Unc-76::GFP
10 ug MRF>H2B::mCherry

Expression in tail muscles/neurons with optimized MRF-152/-136 sites
70 ug Mymk(optMRF-152 + optMRF-136)>Unc-76::GFP
10 ug MRF>H2B::mCherry

Scoring effect of optimized MRF sites + mEbf on ASM expression
100 ug Mymk(optMRF-152 + optMRF-136 + mEbf)>Unc-76::GFP
100 ug Mymk(wt)>Unc-76::mCherry

Scoring wild type Mymk reporter expression to compare to optMRFs+mEbf
100 ug Mymk(wt)>Unc-76::GFP
100 ug Mymk(wt)>Unc-76::mCherry

Protein sequences (related to Figure 2A):

>Ciona EBF (KH.L24.10.v1.A.SL1-1)
MATMAGPQLSGPAVRGWMQTTLVEPMPNGNVGLHRAHFEKQPPNNLRKSNFFHFVLALYDRQGQPVEIER
SAFVGFVENETEIAGEKTNNGIQYRLQLLYHSGVRTEQDVFVRLIDSATKQSITYEGQDKNPEMRRVLLT
HEIMCSRCCDKKSCGNRNETPSDPVVIDRYFLKFFLKCNQNCLKNAGNPRDMRRFQVVVSTTVHVDGHVL
AVSDNMFVHNNSKHGRRARRVDPSEASPTIKAINPAEGWTTGGATVVIVGENFFDGLQVVFGSMVVWSEL
ITQHAIRVQTPPRHLPGVVEVTLSYKNKQFCSGAPGRFVYTALNEPTLDYGFQRLLKTVPRHPGDPERLP
KEIILKRAADVMEAVISRQYAPPSQMPPSAGITPPAPHLAAAPCAPPGSFVPQSASAAMAVAMNGYAAAA
VSSQFGGTPDRFDTGSDSGYSRGNSVSPRNGYSPQTTPHSLNSGSIGSMVGLTTVGAVPAPAPYHCAPSF
NSYSSASGPTFTNMNNTSPGLFSGSGIIPPSPHNGMNPLPSSGTTPGIFSFSPANMISAAKQKSAFAPVH
RPHNSPSPLAPSNGNIALNGYS

>Ciona MRF KH.C14.307.v1.A.SL1-1 (Transcript Variant 2)
MTCISLEELDLSSIFSNSSSYFTSYATNPIMTSQKRTPSRLKRASSDVLLSDTAVSPVSREVSGVLSELD
ELKRCVEGNYIGIDAEKSDISILEELSNMASGCSDSDVAYSSPDSRYGSTGNLTSKTSFGSGMGFRDAGI
GGPVSRGSSLRNPDIRGKRNSIEVKQENEQNPIEFVLESFLNSNETSKPRIHESRVEDIQFTGPPISSIE
PTITFASTEENHNDTVKAMMQYLTETNQMCQENSVPEQIIFSDLNSVSASDSLPSVEELLQIPNEKSRSF
KPDHTAKHQPTLNRPKHFHNQVVNQPHVPILNPELQSFENFSPVINNSLITPTNTFTSNRMMELSPLSDL
HSLSQDEDMDTKMSHYHHTSHPNGHQCLVWACKACKRKTGPHDRRRAATLRERRRLKRVNQAYDALKRCA
CANPNQRLPKVEILRNAITYIYNLQHMLYGDQQSDAKSPETKPETTLSLGETFVSKTEVDSPFYQSDDVR
LTSSRTSSPVESLLESTSSSFIMSDLGDENTQPQDTFPVNLLTDDVTRPSSTTPDVIAVVNEPTTTTEDR
NSSPVTSVSNSDTKGASSLVCLTSIVERID

>Human MYOD1 (CAA40000.1)
MELLSPPLRDVDLTAPDGSLCSFATTDDFYDDPCFDSPDLRFFEDLDPRLMHVGALLKPEEHSHFPAAVH
PAPGAREDEHVRAPSGHHQAGRCLLWACKACKRKTTNADRRKAATMRERRRLSKVNEAFETLKRCTSSNP
NQRLPKVEILRNAIRYIEGLQALLRDQDAAPPGAAAFYAPGPLPPGRGGEHYSGDSDASSPRSNCSDGMM
DYSGPPSGARRRNCYEGAYYNEAPSEPRPGKSAAVSSLDYLSSIVERISTESPAAPALLLADVPSESPPR
RQEAAAPSEGESSGDPTQSPDAAPQCPAGANPNPIYQVL

qPCR primers (relates to Figure 2B):

Gene name	Strand	Sequence (5′ to 3′)	Amplicon size
Human MYMK	Forward	CTTCATGCGTCACGACATCCT	103bp
	Reverse	CCTCTTGGGTTCGTCGAAGT	
Human 18S	Forward	GTAACCCGTTGAACCCCATT	151bp
	Reverse	CCATCCAATCGGTAGTAGCG	

Development • Supplementary information
