# Supplementary material for: A change in cis-regulatory logic underlying obligate versus facultative muscle multinucleation in chordates
Source: Development. 2024 Sep 3;151(20):dev202968. doi: 10.1242/dev.202968 (PMC11441980; doi:10.1242/dev.202968)
Supplement: Supplementary file 1 [file develop-151-202968_review_history.pdf]

A change in cis-regulatory logic underlying obligate versus facultative muscle multinucleation in chordates

Christopher J. Johnson, Zheng Zhang, Haifeng Zhang, Renjie Shang, Katarzyna M. Piekarz, Pengpeng Bi and Alberto Stolfi
DOI: 10.1242/dev.202968

Editor: Karen Sears

Review timeline

Original submission:	16 April 2024
Editorial decision:	6 June 2024
First revision received:	4 July 2024
Accepted:	25 July 2024

Original submission

First decision letter

MS ID#: DEVELOP/2024/202968

MS TITLE: A change in cis-regulatory logic underlying obligate versus facultative muscle multinucleation in chordates

AUTHORS: Christopher J. Johnson, Zheng Zhang, Haifeng Zhang, Renjie Shang, Katarzyna M. Piekarz, Pengpeng Bi, and Alberto Stolfi

I have now received all the referees reports on the above manuscript, and have reached a decision. The referees' comments are appended below, or you can access them online: please go to BenchPress and click on the 'Manuscripts with Decisions' queue in the Author Area.

The overall evaluation is positive and we would like to publish a revised manuscript in Development, provided that the referees' comments can be satisfactorily addressed. Please attend to all of the reviewers' comments in your revised manuscript and detail them in your point-by-point response. If you do not agree with any of their criticisms or suggestions explain clearly why this is so. If it would be helpful, you are welcome to contact us to discuss your revision in greater detail. Please send us a point-by-point response indicating your plans for addressing the referees' comments, and we will look over this and provide further guidance.

Reviewer 1

Advance summary and potential significance to field

In this MS entitled “A change in cis-regulatory logic underlying obligate versus facultative muscle multinucleation in chordates”, Christopher J. Johnson et al. investigate the regulatory mechanisms of the Myomaker (MymK) gene in Ciona robusta to reveal the evolution of multinucleation in muscle myofibers in chordates. In vertebrates, multinucleation has become an obligatory trait that characterizes all skeletal muscles, while in ascidians, multinucleation only affects post-metamorphic muscle in adults, but not axial muscle cells in the tail of the ascidian larva. In this MS, the generation of CRISPR mutants of MRF, the use of reporter expression constructs of MymK, the ectopic expression of MRF and Ebf (both the ascidian proteins as well as the human homologs in a

© 2024. Published by The Company of Biologists under the terms of the Creative Commons Attribution License (https://creativecommons.org/licenses/by/4.0/).

cross-subphyla fashion), the identification of variants of various binding sites identified in the MymK regulatory region, together with the generation of mutant and optimized forms of these binding sites, and RNAseq profiling allow the authors to demonstrate that MymK transcription in post-metamorphic muscle requires the combinatorial activity of MRF and Ebf factors, in a context in which Hex can act as a repressor mechanism of regulation. This finding is an important advance in the field because, for the first time, it demonstrates that cis-regulatory sequence differences in the promoter region of Mymk underlie the different spatiotemporal patterns of its transcriptional activation in tunicates and vertebrates. The significance of this finding is that for the first time it allows to propose two evolutionary models of how the role of Mymk in myoblast fusion might have been regulated in the last common ancestor of olfactores and its divergence between vertebrates and tunicates. Importantly, this MS not only provides fundamental ground knowledge and tools for future studies to test the two evolutionary models of multinucleation in olfactores, but also provides a paradigmatic example that highlights the relevance of studying the evolution of cis-regulatory elements in the investigation of developmental differences between different taxa, also outside the field of tunicates. The MS is very well-written, and despite the complexity and high density of the data, the authors provide a concise and clear description of the results, appropriate figures, supplementary information, an well described methods, which overall should be easily followed by the reader. Therefore, considering the soundness, originality, advances and significance of the MS, I recommend it for publication. Below, I provide only minor comments to be implemented at the discretion of the authors.

Comments for the author

MINOR COMMENTS:

- Throughout the manuscript, the authors use the term “Ciona” to refer to the working model. It would be more appropriate to use “C. robusta" in its place.

- Lines 76-97: The initial two paragraphs within the results section appear primarily introductory and devoid of novel findings. Consequently, I propose to relocate most of this content into the introduction section. Probably, the pictures of figure 1B should be considered the first results of the MS, describing the reporter expression system of MymK used in the next experiments of CRISPR disruption of MRF, followed by the cartoons of the current figure 1A, which are helpful for the reader, although I would include all of them as Figure 1A, and therefore the panel of the current Figure 1C would become 1B, and so on.

- Line 130-137: The results of the Mymk reporter is 0% in the negative control larvae in Figure 1E,F, while it is 7% of transfected larvae in Figure 1G, H. However, the text does not clarify the cause of this difference in the negative controls, and what it is the variability of this reporter system across different assays with different clutches.

- Line 140: a dot is missing“... (Figure S1). We conclude …”

- Line 141-142: “our results suggest that MRF and Ebf co-expression is sufficient for Mymk activation in Ciona.”
In these experiments, I have not been able to find if the reporter construct contains the HES binding site, which the authors later demonstrate can act as a repressor. It is unclear to me, therefore, whether the co-expression of MRF-Ebf would be “sufficient” for the expression of MymK in the presence of HES. Since at this point of the MS, the data for HES have not been reported yet, a possible solution would be to substitute “is sufficient” for “is required”.

- Line 168: a dot is missing “... (Figure 1E-H). This implies …”

- Line 190-191: “Ebf in combination with MRF resulted in a significant reduction of MRF efficacy, appearing to hamper its activation of MYMK”. This result is particularly intriguing and may suggest an interaction between the two proteins or a competition for the binding site of one of the described enhancers (could it be possible?). If so, I please suggest further commenting on this possibility here or/and in the discussion section.

- Line 256-257: I suggest adding “(Figure 4B)” in the sentence “We made mutations predicted to disrupt MRF or Ebf binding to these putative sites in the Mymk>GFP reporter plasmid (Figure 4B),

© 2024. Published by The Company of Biologists under the terms of the Creative Commons Attribution License (https://creativecommons.org/licenses/by/4.0/).

and scored …” to facilitate the reader to easily identify the type of mutations that have been made.

- Line 258-264: The authors report that approximately 20% of mutants show faint expression of GFP, and even the double mutants did not further abolish the GFP expression. This is an interesting observation, but in the text/figures, its interpretation is unclear. Could this be due to mosaicism? Could this be because the mutations are hypomorphic or have low penetrance? Could it be due to natural variability among individuals or differences among embryo clutches in different electroporation assays? Please, if possible, could the authors elaborate in the text possible explanations for these observations.

- Line 323: It is unclear to me how many different combinations are generated for the optimization of the binding sites, and how they are selected. Please, clarify.

- Line 373: Please, reverse the order of “MRF+Ebf” and “MRF-alone” to follow the vertebrate – tunicate logic of the sentences below in this paragraph.

- The discussion is good in general, although there are at least two points in which it could be improved:

o Figure 6 is not easy to follow, specially it is not very helpful to illustrate the two proposed models for the evolution from an ancestral olfactore to vertebrates and ascidians. This figure would gain if it would show the two models in a diagram with phylogenetic trees (for instance, the vertebrate and ascidian current situation in vertical in the middle of the figure, and two trees in each side of the figure illustrating the two possible ancestral situations and the course of events discussed for each model in each side).
o In the discussion, the role of HES is not included in these two models, which makes unclear its potential role in the evolution of vertebrates and ascidians as a repressor regulatory system that can interact with the MRF-Ebf logic and the evolution of myoblast fusion in both taxa. I truly believe that the finding of HES repression is important in the MS, but during the course of the description of the results sometimes it brings many new questions. For instance, how would the optimization of the binding sites behave in the presence of the repressor in the reporter construct? Or what is the expression of HES in C. robusta (as well as in vertebrates)? I understand that many of these new questions might be addressed in future works, but I think that at least including the potential role of HES in the two proposed models of evolution could be informative in the discussion.

Reviewer 2

Advance summary and potential significance to field

In this paper, the authors make an important contribution to our understanding of how regulatory shifts may drive the emergence of new traits within the chordates. In particular, they examine changes in the regulation of Myomixer (mymk) a key gene for myoblast fusion which is expressed in all vertebrate skeletal muscle cells (in vertebrate taxa that have been studied?) but only in adult muscle cells in tunicates, the closest invertebrate relatives of the vertebrates. They identify specific changes in the regulation of Ciona robusta (Cr)-mymk (requirement for co-regulation by Ebf and MRF transcription factors vs. MRF factors alone in vertebrate mymk) that may reflect a broader re-deployment of genes associated with the acquisition of metamorphosis/biphasic life history (including a distinct larval form) in the tunicates from a shared chordate ancestor that likely developed directly (in which larval forms were less distinctive and there was no radical metamorphosis) or vice-versa. Thus their findings also provide insights into more general questions regarding evolutionary basis for the acquisition of metamorphosis or other changes in life history.
In particular they conducted the following experiments.
The study begins with CRISPR LOF studies demonstrating that the sole Ciona ortholog to MRF is required for Cr-mymk expression in adult skeletal muscle.
Strikingly, they were able induce expression of a Cr-mymk reporter in larval muscle by misexpressing Ebf. Additionally, they showed that overexpression of vertebrate Ebf could also

© 2024. Published by The Company of Biologists under the terms of the Creative Commons Attribution License (https://creativecommons.org/licenses/by/4.0/).

induce ectopic Cr-mymk reporter in larval muscle suggesting that trans changes in Ebf coding sequences do not account for shifts in mymk expression in these two clades.
They then showed that expression of Ciona MRF in MYOD1-deficient human myoblasts could induce mymk expression. Intriguingly, co-expression of Ciona Ebf Ciona MRF appears to hamper MRF-dependent activation of mymk expression in these myoblasts indicating that vertebrate and Ciona mymk regulatory elements have conserved binding sites for Ebf that play different functions. Additionally they found that expression of Ebf led to changes in the morphology and gene expression of Ciona larval tail muscles. These results confirmed that Cr-mymk was activated in these conditions along with a number of genes usually associated with adult muscle differentiation leading to partial conversion of laval muscle cells into an adult-like cells (both morphologically and in terms of gene expression).
Interestingly, these converted cells did not undergo fusion indicating that some key part of the fusion gene network was not activated. Additionally, a subset of the ectopically activated genes overlap with genes that are ectopically expressed in cardiopharyngeal-lineage cells due to Ebf misexpression, indicating that tail muscle and cardiopharyngeal progenitor lineages may have distinct lineage-specific factors that alter their response to Ebf over-expression.
They then identified MRF and Ebf binding sites in a Cr-mymk enhancer and showed they were required for reporter activity. They also identified a Hes binding site in this enhancer and showed that it mediates repression, as seen in vertebrate mymk regulation. Lastly, they explored what mutations may have led to divergent mymk expression patterns in tunicates vs. vertebrates. Because vertebrate mymk enhancers do not function in Ciona they explored this question by mutating the Ciona MRF and EBF binding sites to optimize them (increase binding affinity). While optimization of the EBF binding site had no discernible effect, the optimization of MRF binding sites led to ectopic expression in larval tail muscle and in neural lineages indicating that the loss of high affinity MRF binding sites in tunicates may have contributed to loss of mymk expression and myoblast fusion in larval skeletal muscle.

Comments for the author

The experiments in this paper are elegant and the data is highly convincing. The paper is also beautifully written and the discussion is very clear, thorough and informative. No further experiments are needed but I do recommend some minor edits, additional analysis, expanded discussion and changes in their interpretations as listed below.

Comments and suggestions –

It would be helpful if the authors indicated which mymk gene is being referred to throughout the paper (Cr-mymk vs. vertebrate genes) according to agreed upon nomenclature in the tunicate vs. vertebrate communities (lower-case?).
Line 192.
The authors show that co-expression of Ciona Ebf and Ciona MRF appears to hamper MRF-dependent activation of mymk expression in these myoblasts. They interpret this as providing "further support the idea that changes in cis, and not in trans, underlie the differential requirement of Ebf in activating Ciona mymk."
I do not think this is the appropriate interpretation. Instead these results indicate, more specifically, that vertebrate and Ciona mymk regulatory elements may have conserved binding sites for Ebf that play different functions (change in cis). However, it is also possible that changes in the Ciona Ebf protein (trans) mediate this impact on vertebrate mymk expression.
The authors should discuss this possible alternative interpretation.

Line 245 –
The authors show that a subset of genes are differentially activated by ectopic Ebf in larval tail muscle vs. cardiopharyngeal-lineage cells. They interpret this as indicating that "Mymk is just one of several genes that might be preferentially activated in post-metamorphic, multinucleated muscles by a similar MRF-Ebf combinatorial logic in Ciona."
It would be great if they would also mention that this data indicates that tail muscle and cardiopharyngeal progenitor lineages may have distinct lineage-specific factors that alter their response to Ebf over-expression.

Figure 4 –

© 2024. Published by The Company of Biologists under the terms of the Creative Commons Attribution License (https://creativecommons.org/licenses/by/4.0/).

The authors should provide some justification for their strategy for LOF mutation of presumed Ebf and MRF binding sites. A single base pair substitution is usually sufficient and altering multiple base pairs leads to more chance of creating an alternative binding site. Why did they choose to substitute more than 3 or 4 bp?
Line 306 –
For this section I strongly recommend that the authors start off by providing some alternative hypotheses/models regarding changes in cis between vert and tunicate mymk enhancers that may have driven loss of expression in the tunicate larval muscle. As written the logic of this set of experiments is not clear in relation to possible evolutionary changes.
Also, the authors should include some sequence analysis of the vertebrate mymk enhancers to support the hypothesis that more optimal binding sites drive pan-skeletal muscle expression. Do these vertebrate enhancers have highly conserved high affinity MRF binding sites? Maybe a broad alignment across vertebrate species would reveal these sites.
Discussion
The authors have an opportunity to discuss some broader questions that are not specific to mymx regulation and muscle evolution that I encourage them to explore in a revised and expanded discussion. In particular they could touch on
…
1. What is the role of Ebf in vertebrate muscle and how might this have shifted in tunicates? In particular it is interesting that expression of Ciona Ebf and Mrf in vertebrate myoblasts interfered with MRF-dependent expression of mymk. Might Ebf be activating notch and Hes repression? Or is there a better model for this result based on what is currently known about vertebrate mymk regulation?

2. How do their findings inform ideas about life history evolution more broadly? Could changes in binding site affinity provide a generalized mechanism for divergent expression of genes in different life history stages without interfering with ancestral functions/expression?

3. How do their finding touch on questions regarding the specificity of ebox dependent regulation of gene expression? The ability of ebox factors to differentially regulate so many developmental pathways while binding to similar binding sites is a critical question in relation to the structure and function of developmental gene regulatory networks. Some of their data relates to this broader question.

I also have one specific recommendation for the paragraph starting at line 383 – In this paragraph the authors discuss how Ebf and MRF may collaborate to activate Cr-mymk expression. They posit "that MRF and Ebf might act on different steps of Mymk activation in Ciona. In other words, the MRF-Ebf combinatorial logic we have revealed might not be dependent on cooperative binding, as is the case for other examples of cooperativity (Zeitlinger, 2020)."
However, it does not appear that these TFs regulate "different steps" as they both have binding sites in the same enhancer element. Instead it may be that MRF act in a predominant fashion (maybe as a pioneer factor) while Ebf acts secondarily to enhance or permit expression. The authors should modify and expand on this discussion to address some alternative hypotheses.

First revision

Author response to reviewers' comments

We thank the reviewers for their critical reading of the manuscript and for their thoughtful comments. Here are our point-by-point replies.

Reviewer 1 comments:

"Line 130-137: The results of the Mymk reporter is 0% in the negative control larvae in Figure 1E,F, while it is 7% of transfected larvae in Figure 1G, H. However, the text does not

© 2024. Published by The Company of Biologists under the terms of the Creative Commons Attribution License (https://creativecommons.org/licenses/by/4.0/).

clarify the cause of this difference in the negative controls, and what it is the variability of this reporter system across different assays with different clutches."

This is because we were focusing on GFP expression in different territories. In 1E,F, we were focused on larval tail muscle cells. In 1G,H, we were looking for any ectopic GFP expression in Ebf+ cells (mostly the nervous system). We were also conservative in our observations, noting any visible GFP fluorescence even if very weak. The 7% seen in the nervous system in control larvae likely represents weak/leaky/background reporter expression. However, in tail muscles this potential background expression is never visible in control larvae (hence, 0% in 1E,F). We apologize for not explaining this slight difference in the assay, and have clarified and explained it further in the text and figure legend.

"Line 141-142: "our results suggest that MRF and Ebf co-expression is sufficient for Mymk activation in Ciona." In these experiments, I have not been able to find if the reporter construct contains the HES binding site, which the authors later demonstrate can act as a repressor. It is unclear to me, therefore, whether the co-expression of MRF-Ebf would be "sufficient" for the expression of MymK in the presence of HES. Since at this point of the MS, the data for HES have not been reported yet, a possible solution would be to substitute "is sufficient" for "is required"."

The *Ciona Mymk* reporter construct used throughout has the predicted HES site included. We apologize for not clarifying this. It is true, to be more precise our conclusion should be that MRF+Ebf is sufficient for *Mymk* activation *in the absence of any repressive inputs*, such as the proposed Notch/HES repressive mechanism.

In Razy-Krajka et al. 2014, it was shown that Delta-Notch/HES plays a role in temporarily delaying post-metamorphic *Ciona* muscle differentiation in subsets of progenitor cells. More specifically, transient Delta-Notch signaling was shown to suppress differentiation in committed atrial siphon muscle progenitors, allowing for a balance between proliferation and differentiation.

Thus, the post-metamorphic muscles could still grow while also undergoing differentiation, as fully differentiated, multinucleated myofibers cannot proliferate. This would suggest that Delta-Notch/HES might similarly delay *Mymk* activation in committed post-metamorphic progenitors, as fusion would only be enabled in cells undergoing differentiation, not during myoblast proliferation. In fact, it has been shown that Delta-Notch similarly suppresses/delays muscle differentiation and fusion in vertebrates to maintain a population of muscle stem cells (Mourikis et al. 2012). Thus, the role of Delta-Notch/HES in temporarily repressing *Mymk* expression seems to be part of a deeply conserved mechanism for balancing growth/differentiation. We again apologize for glossing over this in the manuscript. These are well established ideas and we have further emphasized them and the supporting citations in the revised text.

Finally, the reason why Ebf and MRF are sufficient to activate *Mymk* in the larval tail muscles could be that there is no evidence that Delta-Notch signaling is active in these cells. We have further updated the text to bring up this fact.

"Line 190-191: "Ebf in combination with MRF resulted in a significant reduction of MRF efficacy, appearing to hamper its activation of MYMK". This result is particularly intriguing and may suggest an interaction between the two proteins or a competition for the binding site of one of the described enhancers (could it be possible?). If so, I please suggest further commenting on this possibility here or/and in the discussion section."

Yes, this was an interesting finding, however, we do not believe there is competition for binding as the DNA-binding preferences of MRFs and EBFs are well characterized and do not overlap, and the DNA-binding domains of tunicate MRF and Ebf are highly conserved, which does not support much divergence in DNA sequence affinity. Our overexpression of human EBF3 in *Ciona* tail muscles also mimicked *Ciona* Ebf overexpression in producing the same ectopic activation of *Mymk* reporter, further arguing against any significant divergence in the very distinct DNA sequence preferences of MRFs and EBFs.

Rather, we hypothesize that Ebf overexpression in these myoblasts likely activates something else

© 2024. Published by The Company of Biologists under the terms of the Creative Commons Attribution License (https://creativecommons.org/licenses/by/4.0/).

that suppresses skeletal muscle differentiation. Perhaps Ebf is promoting a different cell fate, as it is involved in differentiation of neurons and adipocytes in vertebrates. In fact, adipocytes are derived from mesoderm and Ebf2 is a key activator of adipocyte fate (https://www.cell.com/cell-metabolism/fulltext/S1550-4131(13)00047-8). Adipocytes as they are known in vertebrates do not exist in *Ciona*, as far as we know, so perhaps this reflects further subfunctionalization of Ebf specifically in vertebrates.

Or, as suggested by reviewer 2, it's possible that Ebf overexpression is activating Notch signaling, which would activate HES and suppress *Mymk* activation in a way that we do not see in our *Ciona* larval tail muscle. This could be due to timing differences- *Ciona* larvae are assayed before 24 hours have elapsed from fertilization, while cultured mammalian muscles differentiate over the course of several days. Or it could be due to other intrinsic differences in Notch signaling competence.

We have raised these points now in the text and bring up our reasons for thinking this may be due to some indirect repressive effect brought about by Ebf overexpression.

"Line 258-264: The authors report that approximately 20% of mutants show faint expression of GFP, and even the double mutants did not further abolish the GFP expression. This is an interesting observation, but in the text/figures, its interpretation is unclear. Could this be due to mosaicism? Could this be because the mutations are hypomorphic or have low penetrance? Could it be due to natural variability among individuals or differences among embryo clutches in different electroporation assays? Please, if possible, could the authors elaborate in the text possible explanations for these observations."

We do not believe this to be due to mosaicism as the reporter expression was completely lost in *MRF* CRISPR animals (Fig 1D). The most logical explanation is that there may be additional binding sites, still not yet identified, that allow for binding by MRF or another factor and could be responsible for very basal levels of transcription to still take place. We were very conservative in our scoring, scoring even the faintest GFP expression. Indeed we never saw strong GFP expression as with the wildtype or the MRF^{-152} mutant reporters. Unfortunately we did not quantify expression intensity, so this point remains untested. Alternatively, this could represent plasmid reporter "cross-talk" (i.e. transvection), which we frequently observe in *Ciona*. We have elaborated these points in the text now.

"Line 323: It is unclear to me how many different combinations are generated for the optimization of the binding sites, and how they are selected. Please, clarify."

We generated reporter plasmids with "optimized" single MRF and Ebf sites, and the two combined MRF sites. The optimization itself was simply based on editing the flanking sequences of the MRF sites, or the core sequence of the Ebf site, until we got a higher predicted score for each in JASPAR. Therefore these are a first-pass, theoretical "optimization" of these sites. Not absolutely "optimal", but simply "affinity-improved" sites. However, we use the term "optimization" as used by Farley et al. 2015.

In figure S3, we show the predicted JASPAR scores for each "optimized" sequence. Note that because vertebrates have multiple MRF or Ebf paralogs, we had JASPAR predict scores using a wide range of PWMs to get a holistic idea of whether, on average, binding affinity score was really going up according to our optimizations. It also gave us a clear picture that we were likely converting a non-functional MRF site (-152) into one that is probably now a second MRF site after "optimization".

"Figure 6 is not easy to follow, specially it is not very helpful to illustrate the two proposed models for the evolution from an ancestral olfactore to vertebrates and ascidians. This figure would gain if it would show the two models in a diagram with phylogenetic trees (for instance, the vertebrate and ascidian current situation in vertical in the middle of the figure, and two trees in each side of the figure illustrating the two possible ancestral situations and the course of events discussed for each model in each side)."

We thank the reviewer for this suggestion and have now included the two evolutionary models in an expanded Figure 6.

© 2024. Published by The Company of Biologists under the terms of the Creative Commons Attribution License (https://creativecommons.org/licenses/by/4.0/).

"In the discussion, the role of HES is not included in these two models, which makes unclear its potential role in the evolution of vertebrates and ascidians as a repressor regulatory system that can interact with the MRF-Ebf logic and the evolution of myoblast fusion in both taxa. I truly believe that the finding of HES repression is important in the MS, but during the course of the description of the results sometimes it brings many new questions. For instance, how would the optimization of the binding sites behave in the presence of the repressor in the reporter construct? Or what is the expression of HES in C. robusta (as well as in vertebrates)? I understand that many of these new questions might be addressed in future works, but I think that at least including the potential role of HES in the two proposed models of evolution could be informative in the discussion."

We cite previous studies on the role of Notch and HES on *Mymk* expression in vertebrates, but we agree we have not emphasized these points very much in the text. Mostly because the role of HES appears to be exactly shared/conserved/identical between tunicates and vertebrates, while we were focused on what is different between tunicates and vertebrates. As we mention above in an earlier reply, the role of Notch in suppressing muscle differentiation in both tunicates and vertebrates has been established (Razy-Krajka et al. 2014 for *Ciona* work, and many others for vertebrates). The repression of *Mymk* in chick by HES has been shown recently as well (Esteves de Lima et al. 2022). We don't believe HES is binding to the same sites as MRF based on their different preferred sequences (at least according to JASPAR); what scores highly as a predicted MRF site usually scores low (or not at all) as a HES site, and vice-versa. There may be some overlap, but the difference between their sequence preferences seems to lie in the core E-box (CACGTG vs. CAGCTG) and not in the flanking regions we tend to optimize. It would be interesting certainly to "convert" MRF sites into HES sites, but that would be something to address in the future.

As for the pattern of HES in *Ciona,* it was shown in Razy-Krajka et al. (2014) to be expressed in the inner atrial siphon muscle precursors (iASMPs) which are similar to muscle stem cells. This expression was shown to be in response to Delta signals from outer progenitors (oASMPs), which are the first myoblasts to differentiate. This study showed that the Delta/Notch-HES pathway temporarily downregulates MRF in iASMPs and their descendants, which would presumably allow them to remain in a stem cell-like state and proliferate, promoting muscle growth (although this was not definitively shown). Presumably, when Delta/Notch signaling is downregulated, MRF might be reactivated in these cells and they would differentiate (again, this was not directly demonstrated, but proposed). By extension, *Mymk* is likely to be temporarily repressed in by HES in these stem cell-like cells both directly and indirectly (through repression of MRF). In this way, what's happening in *Ciona* would be the same as in vertebrates, so we have not focused on this as much as we were focused on evolutionary differences. We agree that future studies are needed to confirm this proposed conserved role for Delta/Notch-HES in delaying differentiation, *Mymk* activation, and fusion in select myoblasts. We have clarified in the text that these are interesting points but that we believe experimentally addressing them would fall outside the scope of our paper.

Reviewer 2 Comments:

"The authors show that co-expression of Ciona Ebf and Ciona MRF appears to hamper MRF-dependent activation of mymk expression in these myoblasts. They interpret this as providing "further support the idea that changes in cis, and not in trans, underlie the differential requirement of Ebf in activating Ciona mymk." I do not think this is the appropriate interpretation. Instead these results indicate, more specifically, that vertebrate and Ciona mymk regulatory elements may have conserved binding sites for Ebf that play different functions (change in cis). However, it is also possible that changes in the Ciona Ebf protein (trans) mediate this impact on vertebrate mymk expression. The authors should discuss this possible alternative interpretation."

Yes, there is a possibility that the human *MYMK cis*-regulatory region has a cryptic Ebf binding site. Unfortunately the entire *MYMK* surrounding non-coding sequences are over an order of magnitude larger than the *Ciona Mymk* promoter, and testing potential Ebf sites these even in cell culture assays would constitute an entirely new undertaking. Furthermore, the activity of Ebf as a direct repressor has not been well-characterized in the literature. In fact, there are no Google Scholar

© 2024. Published by The Company of Biologists under the terms of the Creative Commons Attribution License (https://creativecommons.org/licenses/by/4.0/).

hits for “Ebf as a repressor”, and only three for “Ebf repressor” (all three refer to repressors of Ebf, not Ebf acting as a repressor). We believe that a more plausible explanation is that Ebf is activating an alternative fate in, or specifically a repressive program that suppresses muscle specification/differentiation when overexpressed in our mammalian cell culture assay (as you suggest further below, perhaps Delta/Notch/HES). The differences between the assays themselves might explain some of the discrepancy. In *Ciona*, our tail muscle assays are focused on the immediate effects of Ebf overexpression since the larva hatches at ~17 hours post-fertilization. In the mammalian cell culture assay, muscle differentiation occurs over three days, and thus we may be seeing far downstream, indirect effects.

“Line 245 - The authors show that a subset of genes are differentially activated by ectopic Ebf in larval tail muscle vs. cardiopharyngeal-lineage cells. They interpret this as indicating that “Mymk is just one of several genes that might be preferentially activated in post-metamorphic, multinucleated muscles by a similar MRF-Ebf combinatorial logic in Ciona.” It would be great if they would also mention that this data indicates that tail muscle and cardiopharyngeal progenitor lineages may have distinct lineage- specific factors that alter their response to Ebf over-expression.”

We apologize for this oversight and have now mentioned this with supporting references.

“1.What is the role of Ebf in vertebrate muscle and how might this have shifted in tunicates? In particular it is interesting that expression of Ciona Ebf and Mrf in vertebrate myoblasts interfered with MRF-dependent expression of mymk. Might Ebf be activating notch and Hes repression? Or is there a better model for this result based on what is currently known about vertebrate mymk regulation?”

EBFs have been shown to be important transcription factors in vertebrate muscles as well. We know that Ebf3 and MyoD1 cooperatively work together to activate *Atp2a1* in mouse diaphragm muscle, (Jin et al. 2014, which we cite in our manuscript). So there is precedent in vertebrates where an MRF-Ebf combinatorial logic could be deployed to regulate muscle type-specific gene expression. Thus it it possible that the combinatorial logic is ancestral, and that his logic has been co-opted (or lost) in different chordate family tree branches to generate muscle subtype diversity.

As for Ebf and Notch, indeed in the *Ciona* siphon muscle lineage Ebf has been shown to activate Delta ligand, HES.b, and other Notch components (Razy-Krajka et al. 2014). This point is now discussed in the revised manuscript.

“2.How do their findings inform ideas about life history evolution more broadly? Could changes in binding site affinity provide a generalized mechanism for divergent expression of genes in different life history stages without interfering with ancestral functions/expression?”

That could indeed be a generalized mechanism, though perhaps more broadly what you mention could involve combinatorial vs. single-factor logic regardless of the underlying molecular mechanism. We are hesitant to suggest the generalizability of our particular findings are entirely novel, as it is similar to previous work on combinatorial logic for tissue-specific expression, it’s just that in this case the different tissues (tail muscle vs. siphon muscle) are separated in the life cycle as well. This is precisely why it is difficult to say whether the tunicate or the vertebrate situation was ancestral, and which is derived, as the last common ancestor likely had these two tissues already separated in space, if not in time.

“3.How do their finding touch on questions regarding the specificity of ebox dependent regulation of gene expression? The ability of ebox factors to differentially regulate so many developmental pathways while binding to similar binding sites is a critical question in relation to the structure and function of developmental gene regulatory networks. Some of their data relates to this broader question.”

We agree this is a fascinating question, but our findings only touch on this through 1) contrast between mutating predicted HES-bound ebox (resulting in more expression), and 2) ectopic activation of the “optimized” reporter in neurons, where potentially neural bHLHs might now

© 2024. Published by The Company of Biologists under the terms of the Creative Commons Attribution License (https://creativecommons.org/licenses/by/4.0/).

be bound to the "optimized" predicted MRF sites, explaining the reporter activation in neurons that we observed. Both are consistent with the idea that slight differences in the core or flanking sequences of eboxes can alter binding preferences by different bHLHs. We do not believe our data provides much insight into this question, though, and we are hesitant to discuss much about this give it is mostly outside the scope of our study.

"I also have one specific recommendation for the paragraph starting at line 383 - In this paragraph the authors discuss how Ebf and MRF may collaborate to activate Cr-mymk expression. They posit "that MRF and Ebf might act on different steps of Mymk activation in Ciona. In other words, the MRF-Ebf combinatorial logic we have revealed might not be dependent on cooperative binding, as is the case for other examples of cooperativity (Zeitlinger, 2020)." However, it does not appear that these TFs regulate "different steps" as they both have binding sites in the same enhancer element. Instead it may be that MRF act in a predominant fashion (maybe as a pioneer factor) while Ebf acts secondarily to enhance or permit expression. The authors should modify and expand on this discussion to address some alternative hypotheses."

This is what we meant by "different steps"- that one might act as a pioneer factor and the other secondarily, i.e. one might open the chromatin, the other recruits RNA polymerase machinery, etc. However, in the absence of more data, especially chromatin profiling data which is outside the scope of our work, we cannot say much about which steps which TFs are affecting. There is some thought that in fact EBF's might be acting as a pioneer factor, and EBF1 is required continuously for chromatin accessibility in pro-B cell lineages: https://www.pnas.org/doi/full/10.1073/pnas.2210595119

We expand a bit on this in the discussion now, also clarifying that by "different steps" we mean things like chromatin accessibility vs. RNA polymerase recruitment/release, etc.

Second decision letter

MS ID#: DEVELOP/2024/202968

MS TITLE: A change in cis-regulatory logic underlying obligate versus facultative muscle multinucleation in chordates

AUTHORS: Christopher J. Johnson, Zheng Zhang, Haifeng Zhang, Renjie Shang, Katarzyna M. Piekarz, Pengpeng Bi, and Alberto Stolfi

I am happy to tell you that your manuscript has been accepted for publication in Development, pending our standard publication integrity checks.

© 2024. Published by The Company of Biologists under the terms of the Creative Commons Attribution License (https://creativecommons.org/licenses/by/4.0/).
